# LCN2 deficiency mitigates the neuroinflammatory damage following acute glaucoma

**DOI:** 10.7150/thno.104752

**Published:** 2025-02-10

**Authors:** Tu Hu, Shuhan Meng, Can Liu, Weizhou Fang, Zhaohua Xia, Yiqun Hu, Jia Luo, Xiaobo Xia

**Affiliations:** 1 Eye Center of Xiangya Hospital, Central South University, Changsha, Hunan, China 410008.; 2 Hunan Key Laboratory of Ophthalmology, Changsha, Hunan, China 410008.; 3 National Clinical Research Center for Geriatric Disorders, Xiangya Hospital, Central South University, Changsha, Hunan, China 410008.; 4 Department of Anatomy and Neurobiology, School of Basic Medical Sciences, Central South University, Changsha, Hunan, China 410013.; 5 Xiangya Medical School, Central South University, Changsha, Hunan, China 410013.; 6 The First Clinical college, Changsha Medical University, Changsha, Hunan, China 410203.

**Keywords:** glaucoma, lipocalin-2, blood-retinal barrier, peripheral immune cells infiltration, innate immune cell response

## Abstract

**Rationale:** Acute high intraocular pressure (IOP) induces retinal ischemia/reperfusion (RI/R) that further initiates neuroinflammatory responses. This event can cause retinal tissue damage and neuronal death, ultimately resulting in irreversible blindness worldwide that lacks effective therapies, validated treatment targets and underlying mechanisms. We sought to explore the potential mechanisms on the causal link between the neuroinflammatory response and neurodegeneration following acute high IOP.

**Methods:** A rat model of RI/R induced by acute high IOP was used to investigate the spatiotemporal profiles of blood-retinal barrier (BRB) disruption, peripheral immune cell infiltration, and innate immune cell response following acute glaucomatous injury. RNA sequencing and *in vivo* transfection with adeno-associated virus (AAV) were used to explore the pathogenic mechanisms of acute high IOP-induced neuroinflammation.

**Results:** Disruption of the inner BRB and infiltration of macrophages and lymphocytes occurred during the early stage after acute high IOP. These events were accompanied by an innate immune response. RNA sequencing revealed that *Lipocalin-2 (Lcn2*) was one of the most significantly up-regulated inflammation-related genes. *Lcn2* knockdown ameliorated inner BRB disruption, peripheral immune cell infiltration, and innate immune cell response, resulting in neuroprotective effects. Furthermore, we found that acute glaucomatous injury triggers high expression of LCN2 in the peripheral serum, which is strongly associated with the severity of the neuroinflammatory response in the retina.

**Conclusions:** A “neuroinflammatory cascade” characterized by breakdown of inner BRB, peripheral immune cell infiltration, and innate immune cell response occurs during the initial stage following glaucomatous injury. We also identified a novel mechanism for LCN2 in acute high IOP-induced neuroinflammation. LCN2 has the potential to serve as a candidate biomarker for predicting the severity of the neuroinflammatory response following acute glaucoma, which may provide new evidence to retinal repair strategies for better visual function recovery at intervention time points and new targets.

## Introduction

The Global Disease Burden report for 2019 indicated that acute glaucoma remains among the leading causes of irreversible blindness worldwide 1. This condition is characterized by a sudden and substantially high intraocular pressure (IOP), severe eye pain, and selective loss of retinal ganglion cells (RGCs), which can progress to visual field loss 2, 3. The pathogenesis of acute glaucoma remains unclear. Clinical lowering of IOP does not stop the progression of secondary nerve damage 4. Therefore, in-depth research into the detailed mechanisms underlying the initiation and progression of RGCs injury in the glaucomatous retina is a critical step toward developing novel therapies to mitigate visual dysfunction.

A rapid increase in IOP in acute glaucoma often results retinal ischemia/reperfusion (RI/R) injury 5. Owing to its inherent vulnerability to ischemic injury, the onset of retinal oxygen-glucose deprivation (OGD) initiates neuronal ischemic necrosis 6, followed by reperfusion, which precipitates deleterious events 7. These events comprise oxidative stress 8, NO release 9, 10, glutamate excitotoxicity 7, and intracellular Ca^2+^ homeostasis disorder 6, all of which contribute to the exacerbation of damage within the retina. Notably, OGD induces neuronal death along with resident glial activation, which leads to the pro-inflammatory cytokines release and attracts peripheral immune cells 11, 12. This process elicits an inflammatory response in the retina 13, triggering various intracellular death mechanisms in retinal neurons via the activation of neuronal membrane receptors 14. Therefore, investigating the critical mechanisms that regulate neuroinflammatory responses in the retina is essential for the treatment and repair of acute high IOP-induced nerve damage and visual dysfunction.

Research has shown that, in response to nerve injuries, damage-associated molecular patterns (DAMPs) can bind to pattern recognition receptors on the membranes of microglia. This interaction induces morphological transformation of microglia and triggers the release of pro-inflammatory cytokines 15-21. It is worth noting that astrocytes and Müller cells, in response to the injury and/or the inflammatory mediators (IL-1α, C1q, TNF, etc.) from the activated microglia, also present as reactive gliosis and production of inflammatory factors 22. Moreover, reactive astrocytes can further promote the pro-inflammatory effects of microglia by activating the complement C3 signaling pathway 23. This reciprocal promotion of pro-inflammatory effects between microglia and astrocytes establishes a positive feedback loop that perpetuates their neurotoxic effects. Our prior investigation revealed that retinal glial cells are activated shortly after the onset of acute high IOP, demonstrating a temporal correlation with the up-regulation of pro-inflammatory genes, such as TNF and IL-1β 24, 25. These findings indicate that retinal glial cells may be instrumental in mediating neuroinflammation following acute glaucomatous injury.

As an immune-privileged site, the retina is shielded from systemic immune surveillance by the blood-retina barrier (BRB) and other immune defense mechanisms 26. However, under ischemic conditions, the BRB undergoes substantial structural and functional alterations. These modifications may enhance the permeability of the BRB, thereby compromising its immune privilege 27, and facilitating the recruitment and infiltration of peripheral immune cells. In addition to their direct participation in the neuroinflammatory response within the retinal tissue, infiltrating peripheral immune cells can activate resident glial cells in the retina 28. Conversely, activated glial cells may release pro-inflammatory mediators, adhesion molecules, and chemokines, which contribute to remodeling/dismantling the BRB and subsequently induce further peripheral immune cell infiltration 29, 30. Based on these findings, we can speculate that the existence of a “neuroinflammatory cascade” that involves resident retinal glial cells, BRB destruction and peripheral immune cells following acute hypoxic-ischemic optic neuropathy. This signaling cascade exacerbates inflammation and contributes to tissue damage. Nevertheless, the current research trajectory seems somewhat fragmented and fails to provide a holistic perspective on this phenomenon. Previous studies have primarily concentrated on the individual aspects of the resident and periphery 11, 17, and the measures implemented have proven inadequate in preventing the progression of the neuroinflammatory response.

Therefore, we presently employed a rat model of ischemia/reperfusion (RI/R) induced by acute high IOP to characterize the neuroinflammation associated with acute glaucoma. Additionally, we investigated the potential factors that may concurrently mediate both the *in situ* and peripheral mechanisms of the retinal immune response to acute glaucomatous injury.

## Materials and methods

### Animals and grouping

Ten-week-old female Sprague-Dawley (SD) rats (220-250 g) were obtained from the Animal Center of Central South University. All animals were maintained under specific pathogen-free conditions with a 12-hours light/dark circle at a temperature of 21 ± 1 °C. Food and water were provided *ad libitum*. The physical conditions of the rats were monitored daily during the experiment. Rats with cataracts, ocular fundus hemorrhages, or infections were excluded.

The rats were randomly divided into four groups: (i) Control group; (ii) RI/R group, rats were treated with anterior chamber compression; (iii) RI/R + LCN2 shRNA group, rats were intravitreally injected of 3 μL of LCN2 shRNA adeno-associated virus (AAV) 28 d before RI/R treatment; (v) RI/R + shNC AAV group, rats were intravitreally injected of 3 μL of shNC AAV 28 d before RI/R treatment.

All animals were treated in accordance with the Association for Research in Vision and Ophthalmology Resolution on the Use of Animals. All animal experiments were reviewed and approved by the Medical Ethics Committee of the Xiangya Hospital of Central South University (approval ID: 2019030519).

### Animal model of RI/R

A rat model of RI/R induced by acute high IOP was established as previously described 31. Briefly, rats were anesthetized with pentobarbital (40 mg/kg; Bayer). The anesthetized rats with dilated pupils (1% tropicamide; Santen) and anesthetized corneas (0.4% oxybuprocaine; Santen) were placed on a heating workbench. A 31-gauge infusion needle connected to a reservoir containing sterile saline was carefully inserted into the anterior chambers of the rats. The reservoir was connected to a pressurizing device (Figure S1A), and the intraocular pressure was slowly elevated to 14.63 kPa (110 mmHg) and maintained for 60 min. After the procedure, tobramycin and dexamethasone ointments were applied to the eyes to prevent corneal drying and infection.

### Cell culture and oxygen‑glucose deprivation reoxygenation (OGD/R) model

BV2 microglia, RAW264.7 macrophages and CTX TNA2 astrocytes were obtained from the ATCC. Cells were cultured in an incubator at 37 °C and 5% CO_2_ in Dulbecco's modified Eagle's medium (DMEM; Gibco) supplemented with 10% fetal bovine serum (FBS; Gibco)) and 1% penicillin streptomycin (Gibco). Cells were maintained via trypsin detachment and passaging daily. We established an oxygen‑glucose deprivation reoxygenation (OGD/R) model for BV2 microglia, RAW264.7 macrophages and CTX TNA2 astrocytes as follows: the original cell culture medium was replaced with glucose-free DMEM (Gibco), and the cells were incubated in an anoxic environment (95% N_2_, 5% CO_2_) for 4 h. The medium was replaced with the original medium, and cells were reoxygenated (21% O_2_, 5% CO_2_) for 3, 6, 12, 24, and 48 h until further examination.

### Acquisition of patient serum

We collected 1 mL of serum from glaucoma patients (with acute angle-closure glaucoma, n = 16) and non-glaucomatous patients (with age-related cataracts, n = 16) at the Xiangya Hospital. Patients with known coronary heart disease, diabetes, or hypertension were excluded. Written informed consent was obtained from all patients. Human studies were approved by the Ethics Committee of Xiangya Hospital (IRB approval number: 202008100) and carried out according to the principles of the Declaration of Helsinki. Serum was quickly collected and immediately frozen and stored at -80 °C until analysis.

### LCN2 shRNA‑AAV transduction

LCN2 shRNA-AAV, shNC-AAV and AAV-GFP were obtained from HANBIO (Shang Hai, China) as follows: Gene ID: NM 170496, interfere sequence: 5′GATCCGCCTCAAGGATAACAACATGTTCAAGAGACGATGTTGTTATCCTTGAGGCTTTTTTG dT3′, serotype: DJ, titer: 1 ×10^12^. Intravitreal injection was administered as described previously 32. Briefly, anesthetized rats were placed on a heated workbench that maintained their body temperature at 35-36 °C throughout the experiments. After corneal anesthesia and pupil dilation with 1% atropine, a puncture was made through the wall of the eye at the posterior angle, 1 mm below the limbus, using a 31-gauge needle under a dissecting microscope. Immediately after, 3μL of LCN2 shRNA-AAV liquid/ shNC-AAV liquid/ AAV-GFP liquid was administered through this aperture using a 34-gauge needle attached to a 5-uL Nanofil Hamilton syringe (Hamilton). After injection, the rats were maintained for 28 d to allow sufficient retinal transduction before subsequent experiments.

### Tissue preparation

For morphological assays, rats were anesthesized and transcardially perfused with saline followed by 4% paraformaldehyde in 0.1 M phosphate buffer (PB, pH 7.4). The eyeballs were enucleated and the cornea, lens, and vitreous body were removed. Some remaining eye cups were post-fixed in 4% PF overnight, immersed in 15% to 30% sucrose solutions at 4 °C for cryoprotection, and then successively sliced into 15 μm thick cross-sections. Some eye cups were post-fixed in 4% PF for 1 h, and the retinae were dissected and cut into four petals in 24-well plates.

For western blotting, qPCR and retinal TNF/IL-1β/CCL2/ICAM-1 ELISA, retinae were dissected from deeply anesthetized rats, weighed, quickly frozen on dry ice, and stored at -80 °C for further homogenization.

For rat serum LCN2 measurements, peripheral blood samples were obtained by removing the eyeballs. Serum was extracted from blood by centrifugation at 3000 rpm for 20 min.

### Retinal whole mount immunofluorescence

The retinal mounts were blocked by incubation in 5% donkey serum (Sigma-Aldrich) containing 0.3% Triton X100 for 2 h at room temperature (RT). To examine endothelial tight junction organization and peripheral immune cells invasion, retinae were incubated with anti-ZO-1 antibody (1:100, Invitrogen, Cat #61-7300), anti-CD45 antibody (1:100, BD bioscience, Cat #550566) and Alexa Fluor 647-labeled isolectin GS-B4 (IB4) from *Griffonia simplicifolia* (1:50 Invitrogen, Cat #I32540) in 5% donkey serum containing 0.1% Triton X-100 for 3 d at 4 °C. Subsequently, Alexa Fluor 488-conjugated donkey anti-mouse (1:1000, Jackson Immunoresearch, Cat #715-545-151) and Alexa Fluor 594-conjugated donkey anti-rabbit (1:1000, Jackson Immunoresearch, Cat #711-585-152) secondary fluorescent antibodies were applied. To examine RGC survival, retinae were incubated with an anti-RBPMS antibody (1:500, GeneTex, Cat #118619), followed by Alexa Fluor 594-conjugated donkey anti-rabbit (1:1000, Jackson Immunoresearch, Cat #711-585-152) secondary fluorescent antibody. Retinae were flat mounted on slides and were covered with a mounting medium containing with 4′,6-diamidino-2-phenylindole (DAPI) (Vector, Cat #H-1800).

### Retinal section immunofluorescence

Retinal sections were blocked by incubation in 5% donkey serum (Sigma-Aldrich) containing 0.3% Triton X100 for 2 h at RT. To examine the functional phenotype of microglia/macrophages, retinal sections were incubated with anti-IBA1 antibody (1:200, Wako, Cat #019-19741), anti-CCR2 antibody (1:200, Proteintech, Cat #16153-1-AP) and anti-MHC-II antibody (1:200, Abcam, Cat #ab23990) in 5% donkey serum containing 0.1% Triton X-100 for 3 d at 4 °C, followed with Alexa Fluor 488-conjugated donkey anti-mouse (1:1000, Jackson Immunoresearch, Cat #715-545-151), Alexa Fluor 594-conjugated donkey anti-rabbit (1:1000, Jackson Immunoresearch, Cat #711-585-152) and Alexa Fluor 647-conjugated donkey anti-goat (1:1000, Jackson Immunoresearch, Cat #705-605-147) secondary fluorescent antibody. To examine complement C3 expression in the inner retina and astrocyte/Müller cells, retinal sections were incubated with an anti-GFAP antibody (1:1000, Cell Signaling Technology, Cat #3670) and an anti-complement C3 antibody (1:200, Abcam, Cat # ab200999), followed by incubation with a secondary fluorescent antibody. To examine LCN2 expression in retinal cells, retinal sections were incubated with an anti-LCN2 antibody (1:200, R&D Systems, Cat #AF1757), anti-GFAP antibody (1:1000, Cell Signaling Technology, Cat #3670), anti-IBA1 antibody (1:200, Wako, Cat #019-19741) and anti-RBPMS antibody (1:200, GeneTex, Cat #118619), and secondary fluorescent antibodies. The sections were covered with a mounting medium containing with DAPI (Vector, Cat #H-1800).

### Tight junction organization grading

To assess tight junction organization, ZO-1 protein continuity at vascular endothelial cell borders was quantified using a semi-quantitative ranking score system based on a scale ranging from 1 to 5, as previously described 33. In brief, a score of 1 was assigned for complete loss of continuous border staining (0%-25% continuous border staining), 2 for 25%-50%, 3 for 50%-75%, 4 for 75%-100%, and 5 for complete continuous border staining. At least four retinae were evaluated in each group, with four images obtained from the mid-periphery regions per retina. Scoring was performed in a masked fashion by three separate evaluators, and the scores were averaged. The frequency of each ranking score were calculated and plotted.

### Measures of retinal vascular permeability

To measure retinal vascular leakage, Evans blue (EB) dye (45 mg/kg body weight) was injected into the tail vein of anesthetized rats and allowed to circulate for 2 h. The vessels were flushed with transcardial perfusion and saline for 3 min. After perfusion, the eyeballs were removed, and the retinae were dissected and weighed to determine dry weight. EB dye was extracted by incubating each retina in 0.3 mL formamide (Sigma-Aldrich) for 18 h at 70 °C. The extracted samples were ultracentrifuged at a speed of 12,000 rpm for 45 minutes at 4 °C. Sixty microliters of the supernatant from each sample were used to measure the absorbance at 620 nm. The concentration of EB dye in the extract was calculated from the standard curve of EB dye in formamide and normalized to retinal dry weight 34, 35.

### Western blotting

Rat retinae or cell samples were homogenized by sonication on ice in radioimmunoprecipitation assay (RIPA) lysis buffer containing a cocktail of protease inhibitors (Sigma-Aldrich). Protein concentration was determined using the BCA method (Thermo Fisher Scientific). Equal amounts of protein (40 µg/lane) from each sample were separated by 4-20% SurePAGE™ Gels (GenScript) and then transferred to PVDF membranes (Millipore). The membranes were blocked with 5% non-fat milk for 2 h at RT, and then were incubated with anti-MHC-II antibody (1:1000, Abcam, Cat #ab23990), anti-Complement C3 antibody (1:1000, Abcam, Cat #ab200999), anti-LCN2 antibody 1:1000 (R&D system, Cat# AF1757), and anti-ZO-1 antibody (1:1000, Invitrogen, Cat #61-7300) overnight at 4 °C. Subsequently, HRP-conjugated secondary antibodies (1:5000, Proteintech) were applied for 2 h at RT.

### ELISA

The levels of TNF, IL-1β, CCL2 and ICAM-1 in retinae at different time points after RI/R injury were evaluated with ELISA kits (CUSABIO, Cat# CSB-E11987r, Cat# CSB-E08055r, Cat# CSB-E07429r, Cat# CSB-E04576r). Retinae were homogenized in PBS, and tissue homogenate supernatants were collected and analyzed according to the manufacturer's instructions. The levels of LCN2 in the serum of rats and patients were quantified using rat LCN2 (CUSABIO, Cat#CSB-E09409r) and human LCN2 ELISA kits (CUSABIO, Cat#CSB-E09408h) according to the manufacturer's instructions.

### RNA isolation and quantitative PCR (qPCR)

RNA was extracted using TRIzol reagent (Invitrogen), and complementary DNA (cDNA) synthesis was performed according to the manufacturer's instructions (Takara). qPCR was performed with a QUANT STUDIO 5 system (Thermo Fisher Scientific) using an SYBR Premix Kit (Takara). The relative expression change of each target mRNA was calculated by the cycle threshold method (ΔΔC_T_ method), with Gapdh as control. Nucleotide sequences of the rat-specific PCR primers were listed in Table S1.

### Linear-regression analysis

Linear-regression analysis was performed to test the correlation between the concentration of LCN2 in rat serum and the expression of TNF/IL-1β/CCL2/ICAM1/MHC-II/C3/ZO-1 in rat retinae. R > 0 represents a positive correlation and R < 0 represents a negative correlation.

### f‑VEP

Anesthetized rats were placed on a heated workbench with three silver electrodes fixed under the skin of the occipital bone (anode electrode), anterior bregma (cathode electrode), and ear (ground electrode). The test eye was exposed to visual stimuli, whereas the opposite eye was covered with an opaque eye shield. Visual stimuli were provided using a multifocal electroretinography recorder (GT-2008V-VI; Gotec). The light stimulation intensity was 10.0 cd·s/m^2^ and the stimulation frequency was 1 Hz. Each f-VEP report was acquired from an average of 64 visual stimuli. The latency of the P2 wave and the amplitude of N2-P2 were measured for further statistical analysis.

### Flow cytometry analysis of retinal immune cells

Rats were anesthetized, their eyeballs were quickly removed, and their retinae were dissected. Two retinae from each rat were pooled as a single sample. Single-cell suspensions were prepared for flow cytometry as described previously 34. Briefly, retinae were diced with a scalpel into < 1 mm pieces and then centrifuged at 450 × g for 5 min at RT. Pelleted tissues were resuspended in 1 ml HEPES-buffered saline (HBSS) with 0.5 mg/mL of Liberase enzyme mix (Roche) and 0.1 mg/mL DNase (Roche) and incubated at 37 °C for 30 min with occasional agitation. After incubation, 1 mL DMEM containing 10% FBS was added to terminate digestion and retinal cells were forcibly strained through a 40 μm nylon mesh cell strainer using the plunger end of a syringe. The strained cell suspension was centrifuged at 450 × g for 5 min at RT and the cell pellet was resuspended in 5 mL of HBSS. The cell suspension was centrifuged at 450 × g for 5 min at RT and the cell pellet resuspended in 100 μL HBSS. The retinal cells were blocked with an anti-CD16/32 antibody (1:50, eBioscience, Cat# 56-0161-82) in PBS containing 20% rat serum for 20 min on ice. The cells were then incubated with Pacific Blue-conjugated anti-CD45 antibody (1:50 volume, BioLegend, Cat #202226) and PE/cyanine7-conjugated anti-CD11b antibody (1:50 volume, BioLegend, Cat#201818) in PBS containing 20% rat serum for 45 min on ice. After incubation, cells were rinsed with PBS and analyzed using an LSRII flow cytometer (BD Biosciences). The data were analyzed using FlowJo software (Tree Star Inc.). Debris and clumps of cells were gated out by forward- and side-scatter analysis in an identical fashion for each group. Immune cell populations were defined by gating the common leukocyte marker CD45 and myeloid lineage marker CD11b. Resident microglia were identified as the CD11b^+^/CD45^med^ population, which differentiated from CD11b^+^/CD45^hi^ myeloid leukocytes. The lymphocytes were sorted into CD11b^neg^/CD45^hi^ cells.

### TUNEL assays

A terminal-deoxy-transferase-mediated dUTP nick end labeling (TUNEL; Roche, #1684795) assay was performed on retinal sections according to the manufacturer's instructions. The sections were incubated with TUNEL reaction solution (including 50 µL Enzyme Solution [TdT] and 450 µL Label Solution [fluorescein-dUTP]) at 37 °C for 1 h and counterstained with DAPI.

### RNA-sequencing

Total RNA was extracted from rat retinae using TRIzol reagent (Invitrogen) according to the manufacturer's instructions. The RNA quality was determined using an Agilent 2100 Bioanalyzer (Agilent Technologies) and quantified using a NanoDrop ND-2000 spectrophotometer (Thermo Fisher Scientific). Only high-quality RNA sample (OD260/280 = 1.8-2.2, OD260/230 ≥ 2.0, RIN ≥ 6.5, 28S:18S ≥ 1.0) was used to construct a sequencing library. RNA purification, reverse transcription, library construction, and sequencing were performed at Shanghai Majorbio Bio-pharm Biotechnology Co., Ltd. (Shanghai, China), according to the manufacturer's instructions (Illumina). The RNA-seq library was prepared using the Illumina Stranded mRNA Prep. Ligation from Illumina using 1μg of total RNA. Briefly, messenger RNA was isolated according to the poly (A) selection method using oligo (dT) beads and then fragmented by fragmentation buffer. Double-stranded cDNA was synthesized using the SuperScript double-stranded cDNA synthesis kit (Invitrogen) with random hexamer primers (Illumina). The synthesized cDNA was subjected to end-repair, phosphorylation and 'A' base addition according to Illumina's library construction protocol. Libraries were selected for cDNA target fragments of 300 bp on 2% low-range ultra-agarose, followed by PCR amplification using Phusion DNA polymerase for 15 PCR cycles. After quantification using Qubit 4.0, the paired-end RNA-seq library was sequenced with a NovaSeq 6000 sequencer (2 × 150bp read length).

### RNA-sequencing data analysis

To identify differentially expressed genes (DEGs) between the two samples, the expression level of each transcript was calculated according to the transcripts per million (TPM) reads method. RSEM was used to quantify the gene abundance. Differential expression analysis was performed using DESeq2. DEGs were filtered with |log2FC| ≧ 1 and with statistical significance (adjusted P-value < 0.05) by R package edgeR (v3.34.0). Principal component analysis (PCA) plots, heat maps, and volcano plots of DEGs were visualized using the R package ggplot2.

### Enrichment analysis of GO and KEGG

Gene Ontology (GO) and Kyoto Encyclopedia of Genes and Genomes (KEGG) enrichment analysis of the DEGs between the different groups were performed to identify significant biological processes and pathways using the R package ClusterProfiler. The enriched results were visualized using the R package ggplot2.

### Gene set enrichment analysis (GSEA)

GSEA was performed based on the GO and KEGG database. Total normalized mRNA expression data were uploaded to the GSEA v3.0 software (http://software.broadinstitute.org/gsea/index.jsp) to generate GSEA enrichment plots.

### Protein-protein interaction (PPI) network analysis

PPI network analysis was performed using String (http://www.string-db.org/, version 12.0). For the network analysis, confidence was set at medium (*p* > 0.4). The string analysis results were visualized using Cytoscape (version 3.7.1).

### Image analysis and quantification

For quantification of immunohistochemical data in retinal sections, six equidistant 160 μm × 135 μm regions of interest (ROI) per section were measured, with two sections per retina. Ten micro-thick Z-stack images were acquired with a 1 µm step size. All images were automatically optimized for brightness and contrast using the ImageJ software (NIH). 3D projection, single plane, and orthogonal views of the Z-stack images were displayed using ImageJ. The number of resident microglia (IBA1^+^CCR2^-^) per infiltrating macrophages (IBA1^+^CCR2^+^) and MHC-II-positive microglia (IBA1^+^CCR2^-^MHC-II^+^) per macrophages (IBA1^+^CCR2^+^MHC-II^+^) in each ROI were counted in a blinded fashion. The relative mean fluorescence intensity of C3 in the retina was calculated using ImageJ software. The colocalized voxels between C3 and GFAP were analyzed using Imaris software (version 9.9, Bitplane).

To quantify the RGC density in the retinal whole mounts, twelve 200 μm x 200 μm images per retina were obtained at 1000, 2000 and 3000 μm (central, middle, and peripheral retina 36) from the optic disk in the superotemporal, superonasal, inferonasal, andinferotemporal quadrants. ImageJ software was used to count the RBPMS-labeled RGCs in each image, and the mean value of 12 images was used to calculate the mean RGC density (RGCs/mm^2^) in each retina.

### Data analysis

Results were presented as the means ± standard deviations (means ± SDs) of data from at least three independent experiments. Statistical analysis was performed using GraphPad Prism (version 8.0). Unpaired two-tailed Student's t-test and one-way analysis of variance (ANOVA), followed by Tukey's multiple comparison test were performed to test for differences between groups. Statistical significance was defined as *p* < 0.05.

## Results

### RI/R injury rapidly disrupts the defensive effects of the inner BRB

**Figure 1:**
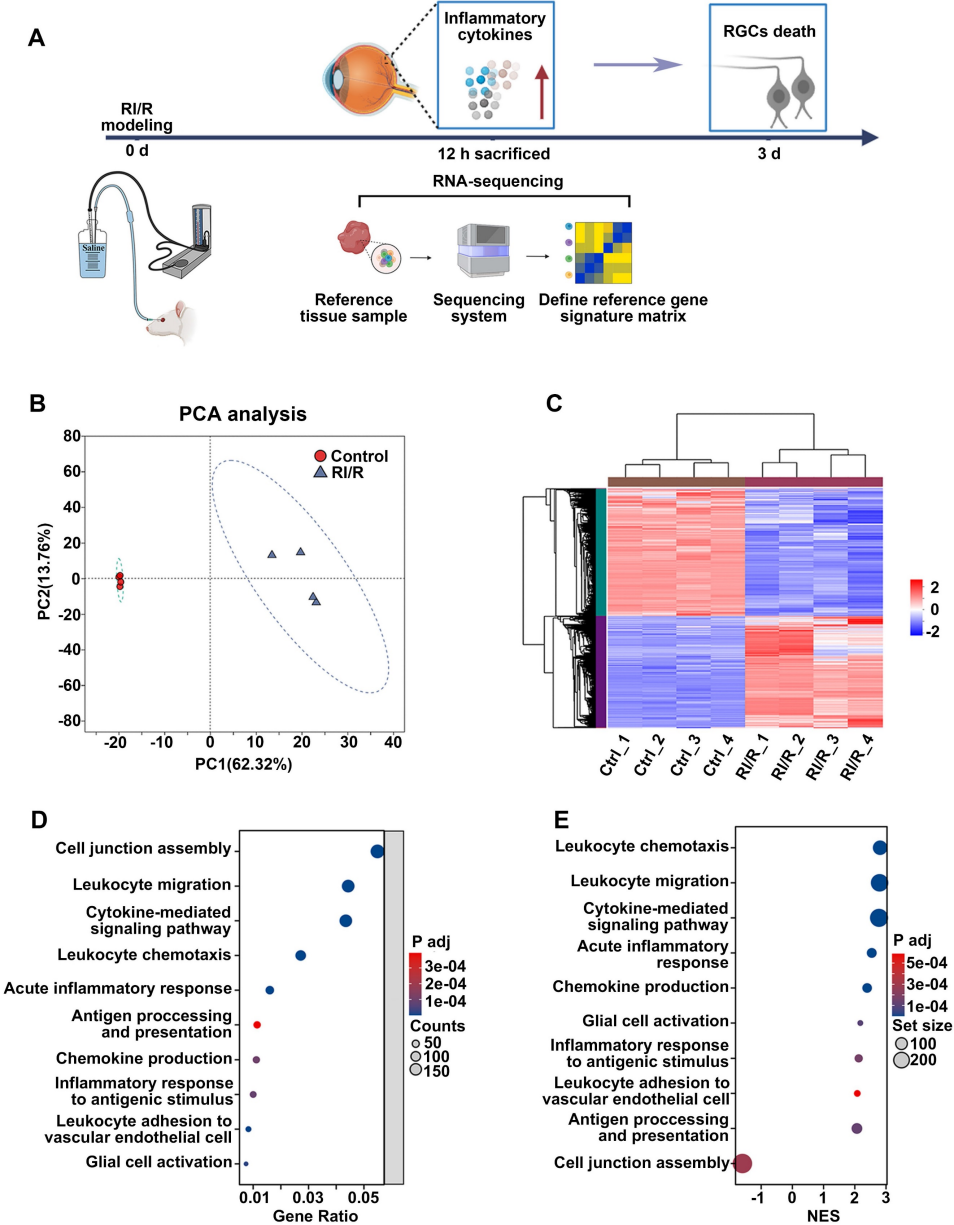
** RNA-sequencing revealed the functional biological processes affected by RI/R injury at the early stages. A:** Diagram revealed that up-regulated levels of inflammatory cytokines occurred earlier than RGCs loss following RI/R injury. RNA-sequencing of rat's retinae was performed at 12 h after RI/R to explore the potential mechanism underlying retinal inflammation. **B**: PCA of retina samples from control and RI/R groups (n = 4 per group).** C**: Heatmap and unsupervised hierarchical clustering of DEGs from control and RI/R retinae. Scale represented z-score values of fragments per kilobase of exon per million (FPKM). **D**: GO Biological Brocess (BP) enrichment of DEGs. **E**: GSEA for enriched GO BP. NES: normalized enrichment score.

ELISA showed that inflammatory cytokines (TNF and IL-1β) were dramatically increased 12 h after RI/R injury (Figure S1B-C), suggesting that an inflammatory response rapidly emerged following RI/R injury. However, immunofluorescence images revealed that no obvious decrease in RBPMS^+^ RGCs emerged until 3 d post-injury (Figure S1D-E). These data indicate that RI/R-induced inflammatory response precedes RGC death, emphasizing that the potential mechanisms of retinal inflammation should be explored in the early phase following RI/R injury. Thus, we performed RNA-sequencing of rat's retinae at 12 h post RI/R injury (Figure 1A). PCA analysis and clustering heatmap suggested significant differences in the transcriptome between RI/R and control retinae (Figure 1B-C). Gene Ontology (GO) enrichment and gene set enrichment analysis (GSEA) results showed that the biological process (BP) term cell junction assembly was significantly down-regulated at 12 h following RI/R injury, meanwhile the BP terms involved in peripheral immune cells infiltration (e.g. chemokine production, leukocyte chemotaxis, leukocyte migration and leukocyte adhesion to vascular endothelial cell), glial cell activation, antigen processing and presentation, inflammatory response (e.g., acute inflammatory response and inflammatory response to antigenic stimulus) and cytokine-mediated signaling pathway were up-regulated (Figure 1D-E). The above bioinformatical analysis predicted that 1) destruction of cell junction, 2) peripheral immune cells infiltration and 3) innate immune cells response occurred soon after RI/R injury.

**Figure 2:**
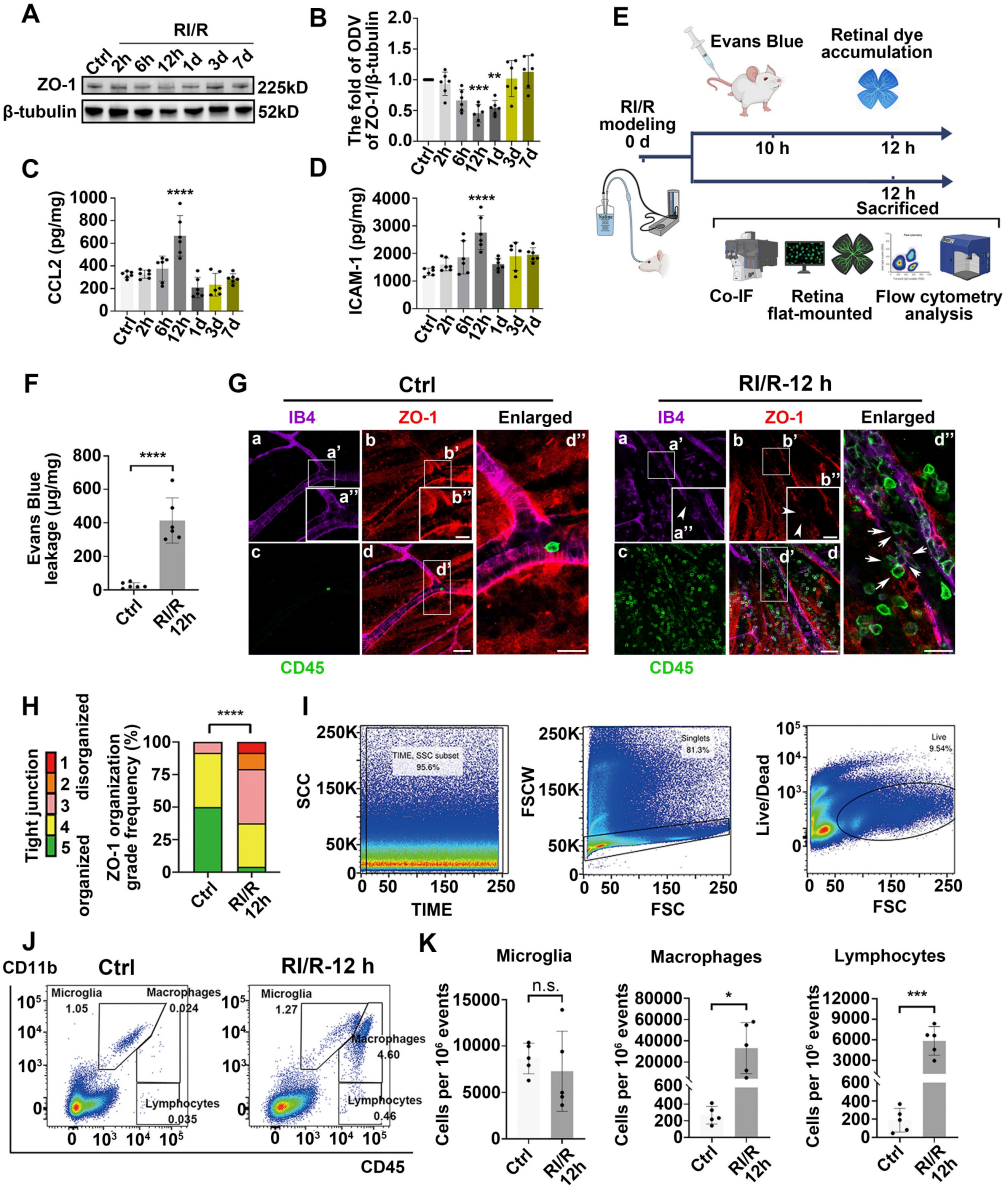
** Early-stage breakdown of the blood-retinal barrier (BRB) and infiltration of peripheral immune cells following RI/R injury. A**: Western blotting showed the changes in ZO-1 expression in retinae following RI/R. **B**: Bar graph depicted the fold of optical density value (ODV) of ZO-1 in each group (n = 6). **C**, **D**: ELISA showed the protein levels of CCL2 and ICAM-1 in rat retinae of each group (n = 6). **E**: Schematic diagram of experimental workflow for detecting the BRB disruption and peripheral infiltration. **F**: Bar graph depicted the retinal Evans blue (EB) dye accumulation in control and RI/R 12 h group (n = 6). **G**: Immunofluorescence images showed IB4 (*green*), ZO-1 (*red*), and CD45 (*magenta*) of superficial vascular regions of normal retinae and RI/R-injured retinae at 12 h after injury. Arrows indicate regions where the vessel is exhibiting disorganization of endothelial tight junction (TJ) complexes and apparent extravasation of CD45^+^ leukocytes. **H**: Histograms represented the evaluation of TJ continuity at endothelial cell borders using a blinded rank (1-5) scoring system. In the graph, green indicated completely continuous, yellow indicated 75% to 100% continuous, pink indicated 50% to 75% continuous, orange indicated 25% to 50% continuous, and red indicated 0% to 25% continuous border staining. For each retina, four images equidistant from the optic disc were averaged with n = 6 retinae for each condition. **I, J**: Representative scatter-graphs showed the flow-cytometric analysis used to quantify immune cell populations in the retina. After gating for single cells, events were gated into CD11b^+^/CD45^med^ cells (resident-microglia), CD11b^+^/CD45^hi^ (myeloid leukocytes) and CD11b^neg^/CD45^hi^ cells (non-myeloid leukocytes, aka lymphocytes). **K**: Flow-cytometric analysis was used to quantify resident microglia, myeloid leukocyte and aka lymphocyte populations in each group (n = 4). Data in **B**-**D**, **F**, **H, K** were represented as mean ± SD; * *p* < 0.05, ***p < 0.001, ****p < 0.0001 (compared with the control group using one-way analysis of variance). Bar = 50 μm in **Ga**-**Gd**, bar = 20 μm in **Ga”**-**Gd”**.

To investigate the dynamic alteration of the inner BRB following RI/R injury, we first examined the tight junctions of the inner BRB by evaluating ZO-1 expression, a central organizer of the tight junction complex 37. Western blotting showed that ZO-1 expression began to decrease 2 h after injury, reached its lowest level at 12 h, and then gradually returned to normal (Figure 2A-B). Immunofluorescent staining of the ZO-1 protein in retinal flat mounts revealed a reduction in ZO-1 protein at the borders by 12 h following RI/R (Figure 2G). The extent of ZO-1 organization at cell borders was graded by a scoring system as previously demonstrated 11, 33. These results indicated a significant disorganization of ZO-1 at 12 h following RI/R (Figure 2H). The EB assay showed that retinal EB dye accumulation drastically increased at 12 h (Figure 2F), indicating an increase in retinal vascular permeability at the early stage following RI/R. However, the breakdown of the inner BRB alone is insufficient to cause the invasion of peripheral immune cells, which also depends on adhesion molecules and chemokines that mediate the accurate location of peripheral immune cells at the injury site 38. ELISA showed that CCL2 (promoting leukocyte adhesion and transmigration across the vascular endothelium 39) and ICAM-1 (triggering the migration of mononuclear macrophages and lymphocytes 40) dramatically increased 12 h after RI/R injury (Figure 2C-D). At the meantime, multi-labeled immunofluorescence showed that a large number of CD45^+^ leukocytes were present in the vessel lumen and retina or were breaking through the lumen wall at 12 h following RI/R (Figure 2G, arrows refer to). To further characterize the changes in retinal immune cell populations, we used flow cytometry to probe all retinal cells for the surface expression of CD45 and CD11b (myeloid markers). A significant accumulation of CD11b^+^/CD45^hi^ (myeloid leukocytes) and CD11b ^neg^/CD45^hi^ cells (non-myeloid leukocytes and lymphocytes) was evident 12 h after RI/R injury, while the number of CD11b^+^/CD45^med^ cells (resident microglia) did not change significantly (Figure 2I-K). These data indicated that a large number of peripheral immune cells rapidly infiltrated into the retinal tissue after the onset of RI/R injury, whereas the resident microglia did not exhibit significant proliferation.

### The innate immune cell response following RI/R injury

Retinal innate immune cells, including microglia, astrocytes, Müller cells, and invading peripheral immune cells, are activated and participate in the immune response at the early stage following acute high IOP-induced RI/R injury 19, 41, 42. Thus, we examined the temporal profiles of morphological and functional phenotypes of these cells in this section of experiments. Antigen presentation by major histocompatibility complex class II (MHC-II) molecules plays a vital role in eliciting an efficient immune response 43. During inflammatory damage to neurodegenerative lesions, the up-regulation of MHC-II serves as a significant indicator of adaptive immune responses 44, 45.

**Figure 3:**
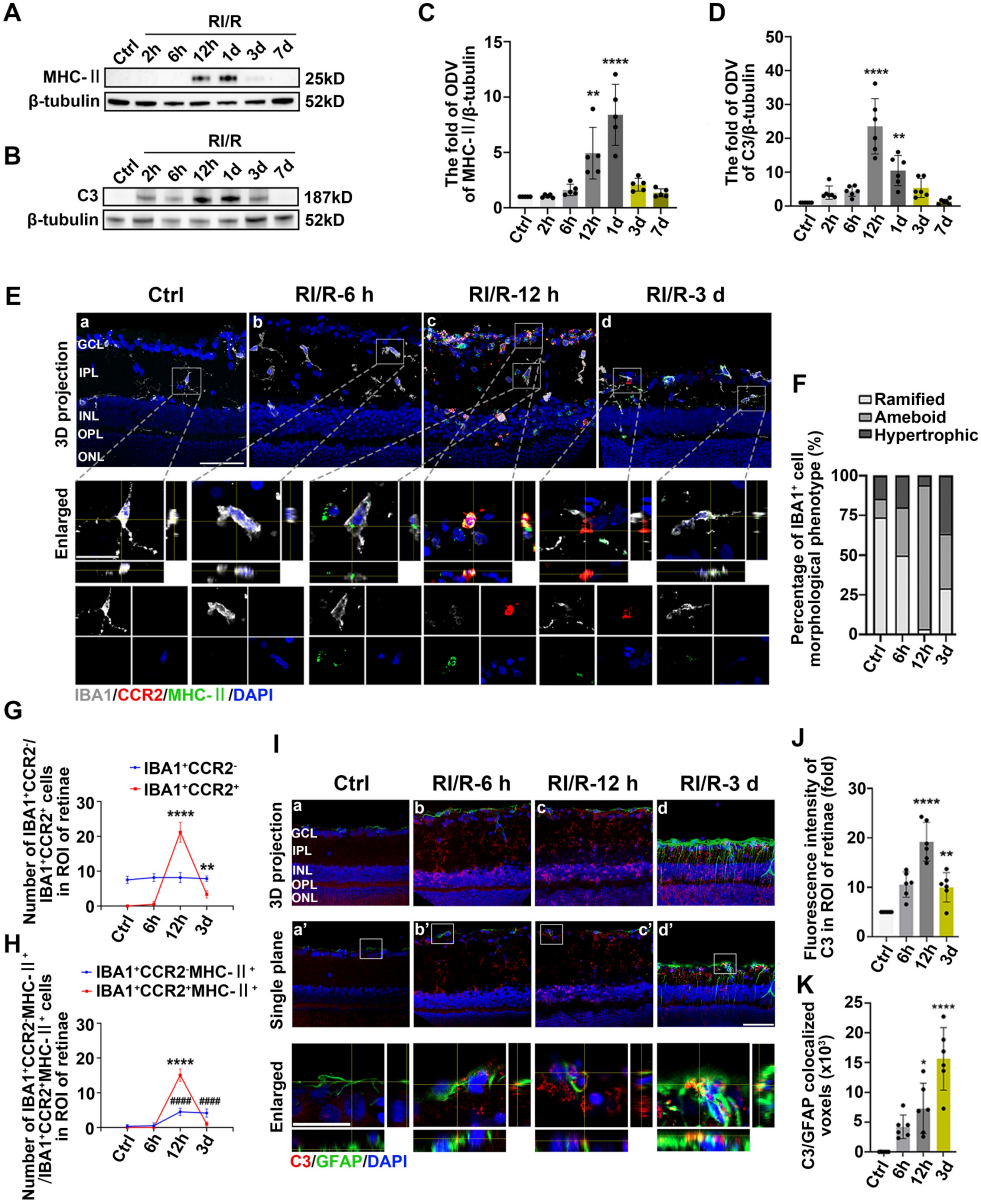
** Innate immune cell response following RI/R injury. A**: Western blotting showed the changes in MHC-II expression in retinae following RI/R. **B**: Western blotting showed the changes in C3 expression in retinae following RI/R.** C**: Bar graphs depicted the fold of ODV of MHC-II in retinae (n = 5). **D**: Bar graphs depicted the fold of ODV of C3 in retinae (n = 6). **E**: Immunofluorescence images showed the morphological alteration of IBA1 (*grey*) positive cells, infiltration of IBA1 (*grey*)/CCR2 (*red*) positive macrophages and distribution of MHC-II (*green*) in rat retinae and in IBA1^+^CCR2^-^ microglia/ IBA1^+^CCR2^+^ macrophages following RI/R. **F**: Bar graph depicted the percentage of morphological phenotypes (ramified, ameboid, hypertrophic) in IBA1 positive cells in each group (n = 6). **G**: Bar graphs depicted the number of IBA1^+^CCR2^-^ and IBA1^+^CCR2^+^ cells in each group (n = 6).** H**: Bar graphs depicted the number of IBA1^+^CCR2^-^MHC-II^+^ and IBA1^+^CCR2^+^MHC-II^+^ cells in each group (n = 6). **I**: Immunofluorescence images showed the morphological alteration of GFAP (*green*) positive cells, the distribution of C3 (*red*) in rat retinae, and co-staining of GFAP and C3 (*orange*) following RI/R. **J**: Bar graph depicted the fluorescent intensity of C3 in inner retinae in each group (n = 6). **K**: Bar graph depicted the colocalized voxels of C3/GFAP staining in inner retinae in each group (n = 6). Data in** C, D, G, H, J, K** were represented as mean ± SD; *p < 0.05, **p < 0.01, ****p < 0.0001, ####*p* < 0.0001 (compared with the control group using one-way analysis of variance). ####*p* < 0.0001 in **G**, **H** for IBA1^+^CCR2^-^MHC-II^+^ cells comparison; **p < 0.01, ****p < 0.0001 in **G**, **H** for IBA1^+^CCR2^+^ and IBA1^+^CCR2^+^MHC-II^+^ cells comparison. Bar = 50 μm in **Ea**-**Ed**, **Ia-Id, Ia'**-**Id',** bar = 20 μm in** enlarged images**. GCL: ganglion cell layer, IPL: inner plexiform layer, INL: inner nuclear layer, OPL: outer plexiform layer, ONL: outer nuclear layer, ROI: region of interest.

**Figure 4:**
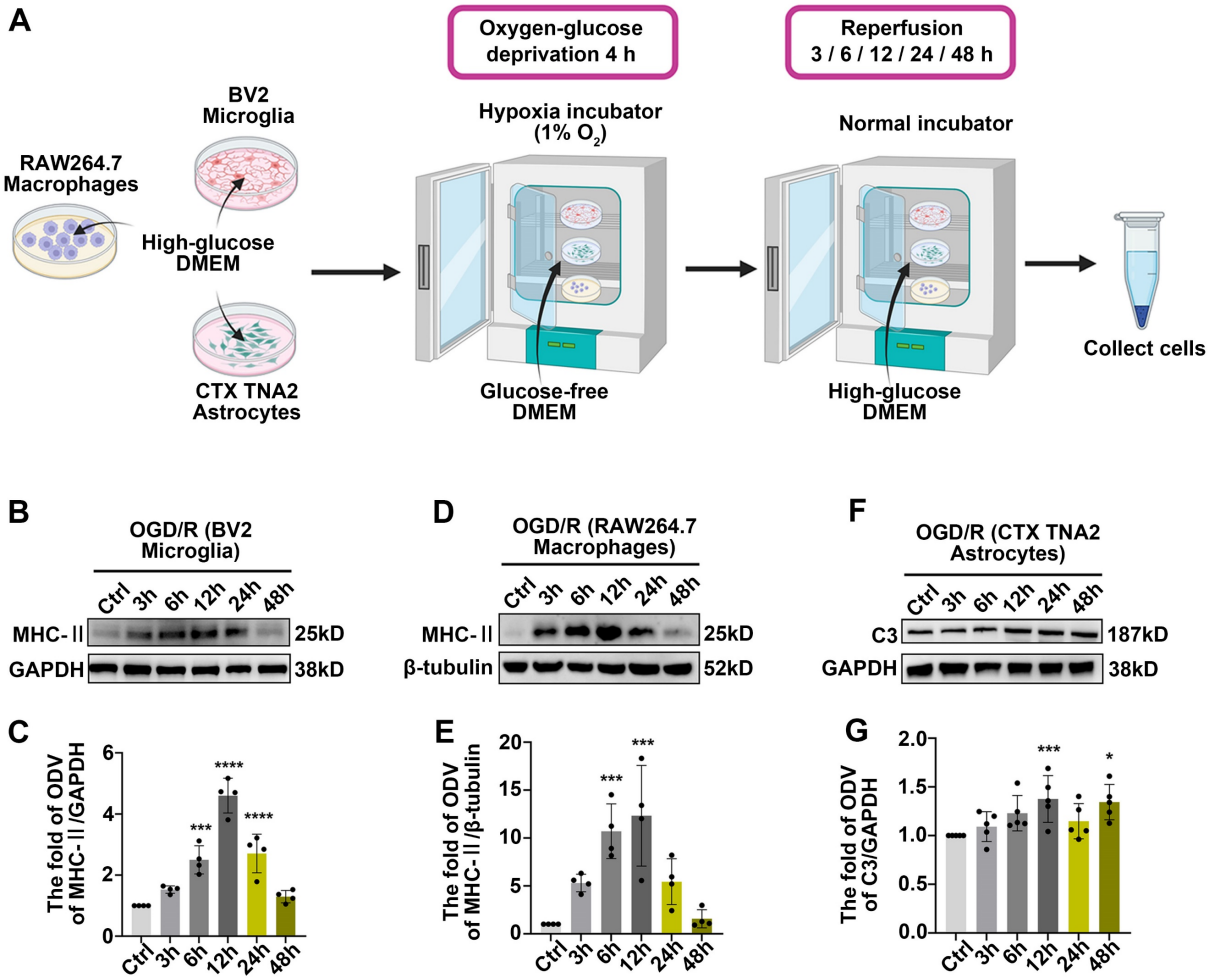
** Innate immune cell response after OGD/R injury. A**: Diagram of OGD/R *in vitro* model. **B**: Western blotting showed the changes in MHC-II expression in BV2 microglia following OGD/R. **C**: Bar graphs depicted the fold of ODV of MHC-II in BV2 microglia (n = 4). **D**: Western blotting showed the changes in MHC-II expression in RAW264.7 macrophages following OGD/R. **E**: Bar graphs depicted the fold of ODV of MHC-II in RAW264.7 macrophages (n = 4). **F**: Western blotting showed the changes in C3 expression in CTX TNA2 astrocytes following OGD/R. **G**: Bar graphs depicted the fold of ODV of C3 in CTX TNA2 astrocytes (n = 5). Data in** C, E, G** were represented as mean ± SD; ***p < 0.001, ****p < 0.0001 (compared with the control group using one-way analysis of variance).

This phenomenon suggests that under pathological conditions, microglia or other antigen-presenting cells (APCs) may be involved in triggering specific immune responses, thereby exacerbating inflammation and influencing the disease progression 46. Based on these evidences, MHC-II, IBA1 (a pan marker of microglia and macrophage 47, 48) were introduced to investigate the effects of innate immune cells in antigen presentation following acute glaucomatous injury. Immunofluorescence images showed that most IBA1^+^ cells in healthy retina appeared as ramified shapes, characterized by a small soma and highly branched processes (Figure S2). This morphological feature enables microglia to detect subtle changes in the surrounding microenvironment 49. Following RI/R injury, IBA1^+^ cells transformed rapidly from a ramified to an ameboid shape, indicating drastic activation of retinal microglia and invading macrophages (Figure S2; Figure 3E-F). Western blotting assays revealed that the protein levels of MHC-II in retinal tissues significantly increased and peaked within 12 h following RI/R injury (Figure 3A, C). To further explore whether microglia and macrophages represented by IBA1^+^ cells are involved in antigen presentation, CCR2 (a special marker of monocyte-derived macrophage 50) was utilized in the following multi-labeled immunofluorescence. We found that IBA1^+^CCR2^+^ cells (macrophage), rather than IBA1^+^CCR2^-^ cells (resident microglia), rapidly increased following RI/R injury (Figure 3E, G). At the meantime, both IBA1^+^CCR2^+^MHC-II^+^ cells and IBA1^+^CCR2^-^MHC-II^+^ cells also increased (Figure 3E, H). Our *in vitro* data showed an initial increase, followed by a gradual decrease, in the protein level of MHC-II under separate cultivation of BV2 microglia and RAW264.7 macrophages following OGD/R (Figure 4B-E). These data implied that retinal microglia and invaded macrophages might participate in antigen presentation soon after RI/R injury.

Acute experimental complement cascade blockade can rescue tissues and improve stroke-induced functional outcomes 51, highlighting the crucial effect of the complement system on the secondary injury within ischemic and traumatic CNS diseases. Among the various complement components, complement C3 stands out for its opsonization and adhesion properties, which profoundly influence subsequent phagocytosis and antigen-antibody interactions, establishing it as a critical element of the immune response 52. Prior research identified astrocytes as the primary cell type expressing C3 in the aftermath of ischemic nerve injuries 53. Therefore, we utilized C3 and GFAP (a pan marker of astrocytes, and Müller cells) to assess the effects of astrocytes and Müller cells involvement in complement activation. Western blotting assays *in vivo* demonstrated an initial increase, followed by a gradual decrease, in C3 protein levels after RI/R injury (Figure 3B, D). Quantitative analysis also showed that the fluorescence intensity of C3 staining in the inner retina significantly increased at 12 h after injury and gradually decreased thereafter (Figure 3I-J). Colocalization analysis revealed that C3^+^/GFAP^+^ (a pan marker of astrocytes and Müller cells) colocalized voxels also increased at 12 h and remained elevated at 3 d following RI/R (Figure 3I, K). Under separate cultivation conditions, the protein levels of C3 in CTX TNA2 astrocytes significantly increased within 12 h following OGD/R (Figure 4F-G). This suggests that reactive astrocytes and/or Müller cells are involved in complement activation early after RI/R injury. Notably, complement C3 facilitates blood-nerve barrier (BNB) leakage and peripheral infiltration in CNS diseases 23, 54, 55.

Thus far, it can be postulated that the reciprocal promotion among the breakdown of the inner BRB, infiltration of peripheral immune cells, and immune responses mediated by innate immune cells may be significant causes of the exacerbation of neuroinflammation early following acute glaucomatous injury.

### LCN2 might have an intimate relationship with the inner BRB breakdown, the peripheral immune cell infiltration, and the activation of retinal glial cells following RI/R injury

**Figure 5:**
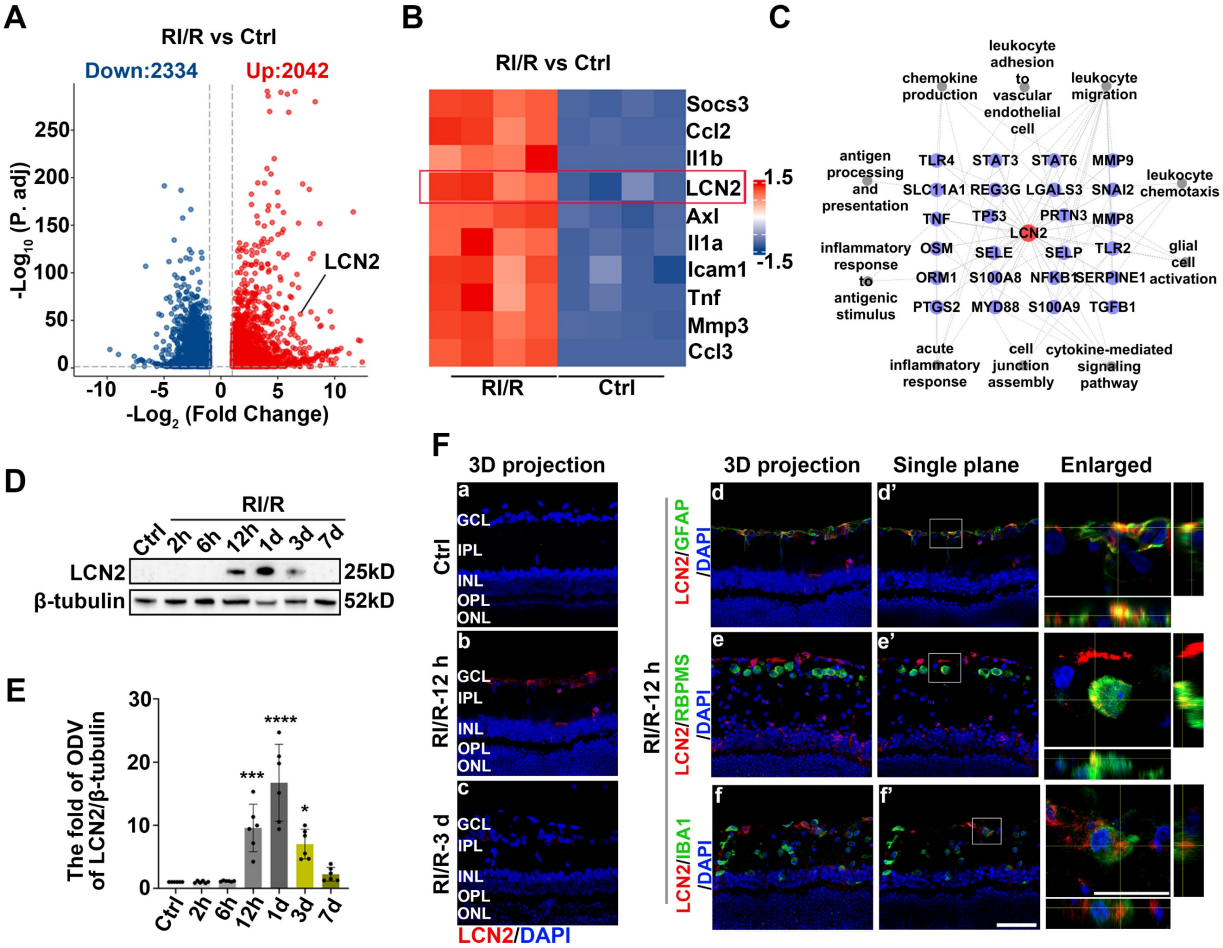
** LCN2 rapidly up-regulated following RI/R injury and might have an intimate relationship with BRB disruption, peripheral infiltration, and activation of retinal glial cells. A**,** B**: Volcano plot and heatmap showed that LCN2 was one of the highly up-regulated inflammation-related genes at 12 h after RI/R **C**: The interaction diagram depicted that LCN2 interacted with proteins belonging to enriched GO terms following RI/R. **D**: Western blotting showed the changes in LCN2 expression in retinae following RI/R. **E**: Bar graph depicted the fold of ODV of LCN2 in each group (n=6). **F**: Immunofluorescence images showed the distribution of LCN2 (*red*) in rat retinae and colocalization of LCN2 with GFAP/RBPMS/IBA1 (*green*) following RI/R injury. Data in** E** were represented as mean ± SD; *p < 0.05, ***p < 0.001, ****p < 0.0001 (compared with the control group using one-way analysis of variance). Bar = 50 μm in **Fa-Ff, Fd'-Ff'**, bar = 20 μm in** enlarged images**. GCL: ganglion cell layer, IPL: inner plexiform layer, INL: inner nuclear layer, OPL: outer plexiform layer, ONL: outer nuclear layer.

The present study provides evidence that the early stages following acute glaucomatous damage are characterized by the simultaneous occurrence of inner BRB breakdown, peripheral immune cell infiltration, and immune response by innate immune cells. These events appear to synergistically reinforce each other 27, 29, 30, thereby leading to the initiation of “neuroinflammatory cascade” that persists. Therefore, we explored the key mechanisms that could simultaneously regulate these above pathological events, and then suppress them accordingly. Our RNA sequencing analysis revealed that RI/R induced the up-regulation of 2334 genes and down-regulation of 2042 genes in the rat retina (Figure 5A). *Lipocalin 2* (*Lcn2*) was one of the most significantly up-regulated inflammation-related genes 12 h post-RI/R injury (Figure 5B). Through literature review, LCN2 is a member of the secreted lipocalin protein family and plays a crucial role in promoting neuroinflammatory response 56.

Several studies have reported that LCN2 regulates diverse cellular processes, including migration, invasion, and polarization of immune cells 57. Using protein-protein interaction (PPI) network analysis, we found that LCN2 interacted with molecules belonging to the enriched BP term in a previous GO enrichment analysis (Figure 5C). These results suggest that LCN2 may participate in the above multiple events following RI/R and may serve as a potential target involved in the mechanisms that may simultaneously regulate inner BRB breakdown, peripheral immune cell infiltration, and immune responses of innate immune cells following RI/R injury. Western blotting analysis revealed that LCN2 expression was detectable at 12 h and peaked at 1 d following RI/R (Figure 5D-E). Immunofluorescence staining revealed that increased LCN2 was distributed in the inner layers of rat retinae and primarily colocated with GFAP, while exhibiting a lesser degree of colocalization with both RBPMS and IBA1 (Figure 5F). This implies that elevated LCN2 levels induced by RI/R may be derived from multiple cell types, mainly astrocytes and/or Müller cells. Notably, the time course of LCN2 up-regulation induced by RI/R injury was consistent with BRB disruption (Figure 2A-B, F-H), infiltration of peripheral immune cells (Figure 2G, I-K), immune response by innate immune cells (Figure 3), and an increase in pro-inflammatory cytokines (Figure S1B-C; Figure 2C-D).

### Suppressing LCN2 ameliorated the disruption of inner BRB and the infiltration of peripheral immune cells following RI/R injury

**Figure 6:**
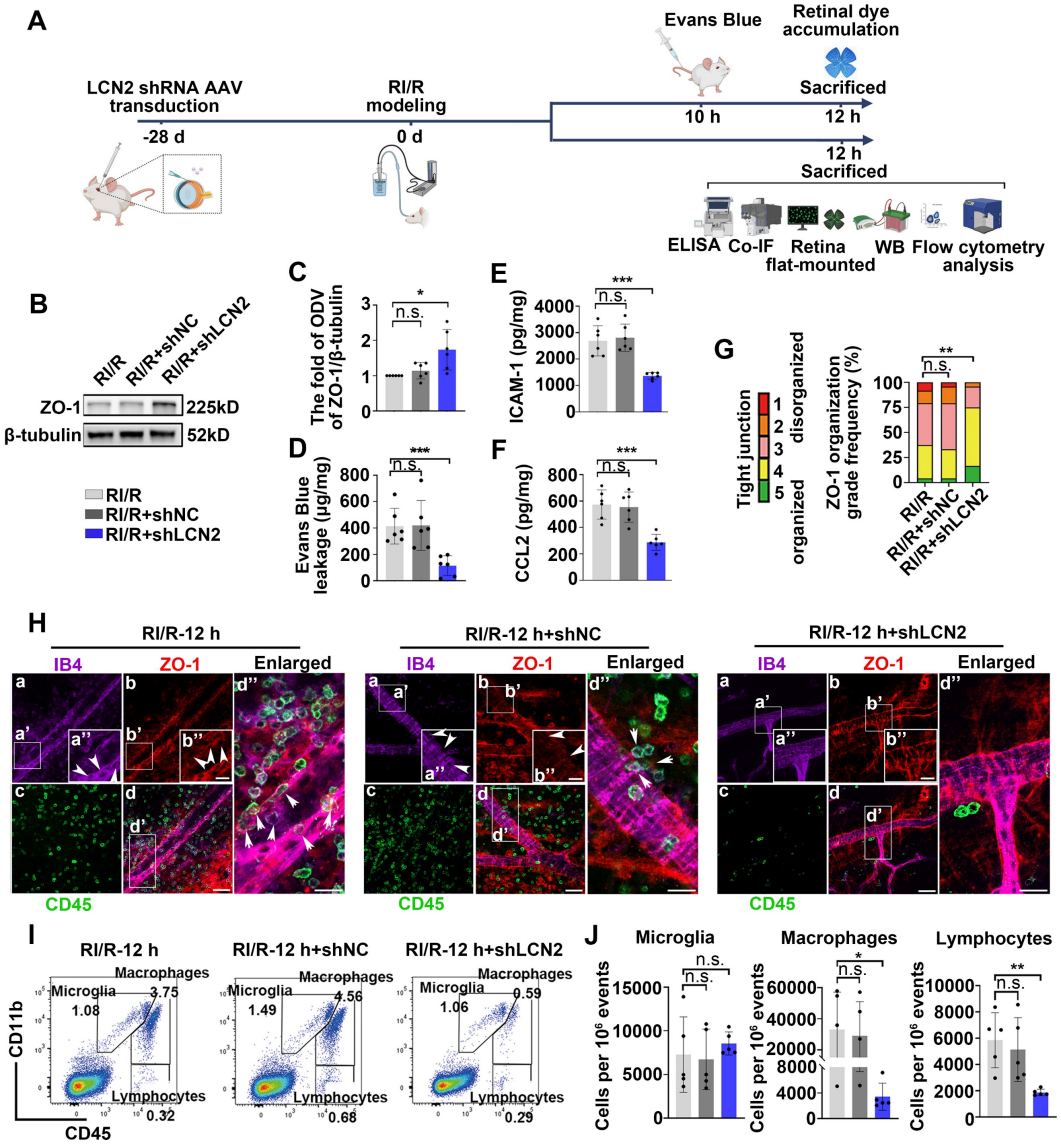
** LCN2 shRNA-AAV treatment ameliorated the disruption of BRB, and infiltration of peripheral immune cells. A**: Diagram presents the research workflow. **B:** Western blotting showed the changes in ZO-1 expression following LCN2 shRNA-AAV treatment, compared to the RI/R group. **C**: Bar graph depicted the fold of ODV of ZO-1 in each group (n = 6). **D**: Bar graph depicted the retinal Evans blue dye accumulation following LCN2 shRNA-AAV treatment, compared to the RI/R group (n = 6). **E**, **F**: ELISA showed the protein level of CCL2 and ICAM-1 in rat retinae of each group (n = 6). **G**: Histograms represent the evaluation of TJ continuity at endothelial cell borders using a blinded rank (1-5) scoring system. In the graph, green indicates completely continuous, yellow indicates 75% to 100% continuous, pink indicates 50% to 75% continuous, orange indicates 25% to 50% continuous, and red indicates 0% to 25% continuous border staining. For each retina, four images equidistant from the optic disc were averaged with n = 6 retinae for each condition. **H**: Immunofluorescence images showed IB4 (*green*), ZO-1 (*red*), and CD45 (*magenta*) of superficial vascular regions of RI/R-injured retina, RI/R+LCN2 shRNA treated retinae and RI/R+shNC treated retinae at 12 h after injury. Arrows indicate regions where the vessel is exhibiting disorganization of endothelial TJ complexes and apparent extravasation of CD45+ leukocytes. **I**: Representative scatter-graphs showing the flow-cytometric analysis used to quantify immune cell populations in the retina. After gating for single cells, events were gated into CD11b^+^/CD45^med^ cells (resident-microglia), CD11b^+^/CD45^hi^ (myeloid leukocytes) and CD11b^neg^/CD45^hi^ cells (non-myeloid leukocytes, aka lymphocytes). **J**: Flow-cytometric analysis was used to quantify resident microglia, myeloid leukocyte and aka lymphocyte populations in each group (n = 5). Data in **C**-**F, J** were represented as mean ± SD; *p < 0.05, **p < 0.01, ***p < 0.001, n.s.: no significance, (compared with the RI/R group using Student t-test analysis of variance). Bar = 50 μm in **Ha**-**Hd**, bar = 20 μm in **Ha”**-**Hd”**.

To investigate the potential involvement of LCN2 in the neuroinflammation following RI/R injury, we utilized LCN2 shRNA-AAV (adeno-associated virus) to knock down retinal LCN2. Western blotting analysis revealed that the high expression of LCN2 in the whole retina was suppressed following RI/R injury (Figure S3A-B). Further western blotting assays revealed that the protein levels of ZO-1 were significantly higher in the RI/R+LCN2 shRNA group than that in the RI/R group and the RI/R+shNC group (Figure 6B-C). The accumulation of retinal EB dye was significantly reduced in the RI/R+LCN2 shRNA group compared to that in the RI/R group and the RI/R+shNC group (Figure 6D). Furthermore, ELISA showed that *Lcn2* knockdown significantly decreased the RI/R-induced up-regulation of CCL2 and ICAM-1 levels (Figure 6E-F). Immunofluorescence experiments demonstrated that the knockdown of LCN2 reversed the reduction in ZO-1 at the borders and improved ZO-1 disorganization, as indicated by the scoring system (Figure 6G-H). These results suggested that *Lcn2* knockdown reduced inner BRB disruption and vascular leakage after RI/R injury. In addition, immunofluorescence images showed that the number of CD45^+^leukocytes present in the vessel lumen, within the retinal tissue, or within the lumen wall were significantly lower in the RI/R+LCN2 shRNA group than in the RI/R group and the RI/R+shNC group (Figure 6H). Flow cytometry analysis demonstrated that the increase in the number of infiltrating monocyte/macrophages (CD11b^+^/CD45^hi^) and lymphocytes (CD11b^neg^/CD45^hi^) induced by RI/R injury was attenuated in the RI/R+LCN2 shRNA group (Figure 6I-J). These findings indicated that *Lcn2* knockdown alleviated inner BRB disruption and the infiltration of peripheral immune cells.

### *Lcn2* knockdown suppressed the innate immune cell response following RI/R injury

**Figure 7:**
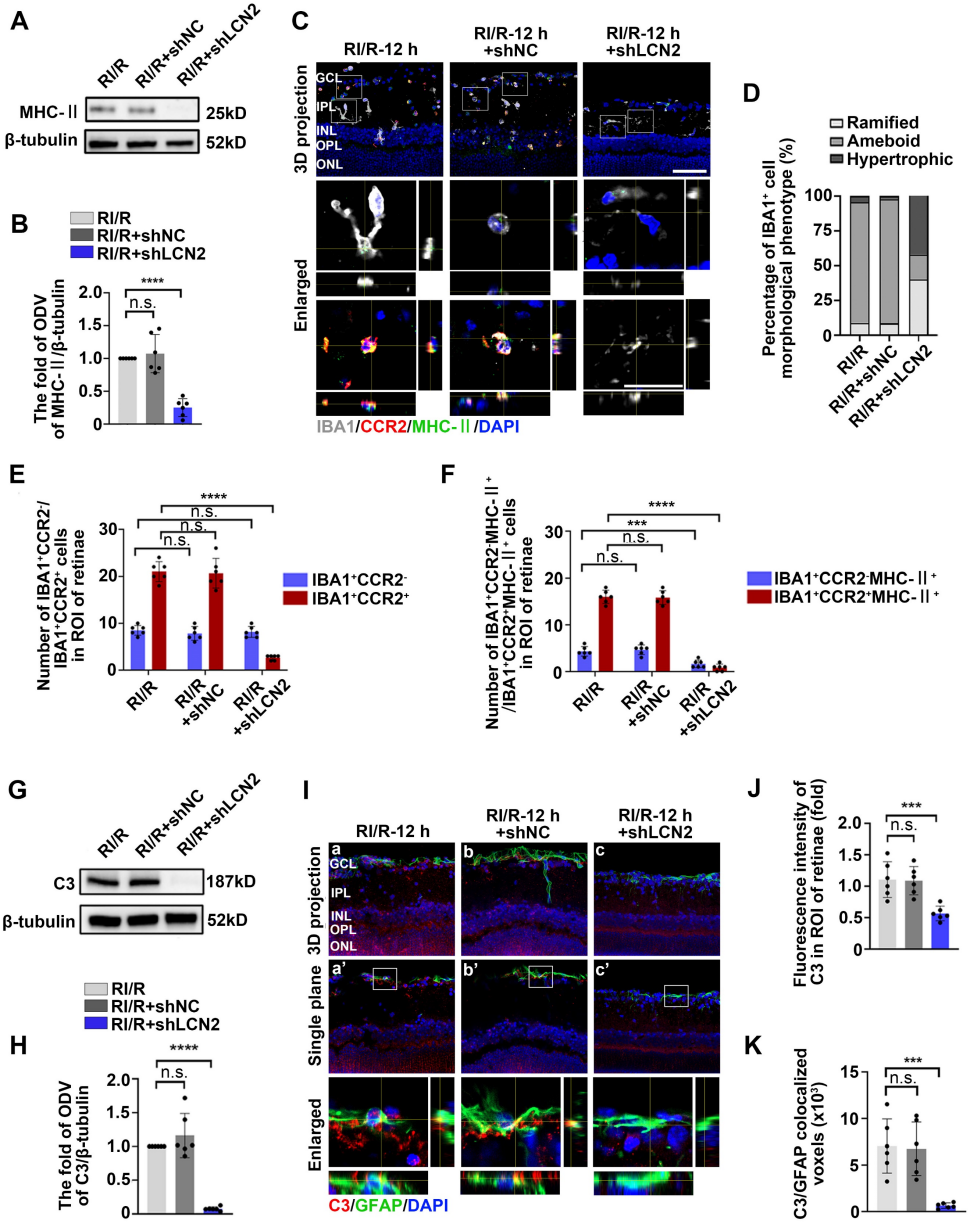
** LCN2 shRNA-AAV treatment suppressed the innate immune cell response. A**: Western blotting showed the changes in MHC-II expression following LCN2 shRNA-AAV treatment, compared to the RI/R group. **B**: Bar graphs depicted the fold of ODV of MHC-II in each group (n = 6). **C**: Immunofluorescence images showed the morphological alteration of IBA1 (*grey*) positive cells, infiltration of IBA1 (*grey*)/CCR2 (*red*) positive macrophages and distribution of MHC-II (*green*) in rat retinae and in IBA1^+^CCR2^-^ microglia/IBA1^+^CCR2^+^ macrophages following RI/R. **D**: Bar graph depicted the percentage of morphological phenotypes (ramified, ameboid, hypertrophic) in IBA1 positive cells in each group (n = 6). **E**: Bar graphs depicted the number of IBA1^+^CCR2^-^ and IBA1^+^CCR2^+^ cells in each group (n = 6).** F**: Bar graphs depicted the number of IBA1^+^CCR2^-^MHC-II^+^ and IBA1^+^CCR2^+^MHC-II^+^ cells in each group (n = 6). **G**: Western blotting showed the changes in C3 expression following LCN2 shRNA-AAV treatment, compared to the RI/R group. **H**: Bar graphs depicted the fold of ODV of C3 in each group (n = 6). **I**: Immunofluorescence images showed the morphological alteration of GFAP (*green*) positive cells, the distribution of C3 (*red*) in rat retinae, and co-staining of GFAP and C3 (*orange*) following LCN2 shRNA-AAV treatment. **J**: Bar graph depicted the fluorescent intensity of C3 in inner retinae in each group (n = 6). **K**: Bar graph depicted the colocalized voxels of C3/GFAP staining in inner retinae in each group (n = 6). Data in **B**, **E**, **F**, **H**, **J**, **K** were represented as mean ± SD; ***p < 0.001, ****p < 0.0001, n.s.: no significance, (compared with the RI/R group using Student t-test). Bar = 50 μm in **Ca**-**Cc**,** Ha**-**Hc**, **Ha'**-**Hc'**, bar = 20 μm in** enlarged images**. GCL: ganglion cell layer, IPL: inner plexiform layer, INL: inner nuclear layer, OPL: outer plexiform layer, ONL: outer nuclear layer, ROI: region of interest.

Western blotting assays revealed that MHC-II protein levels were decreased in the RI/R+LCN2 shRNA group compared to the RI/R group and RI/R+shNC group (Figure 7A-B). Immunofluorescence images and analysis demonstrated that the proportion of IBA1^+^ cells with ameboid morphology was notably decreased in the RI/R+LCN2 shRNA group, while the fractions of ramified IBA1^+^ cells was increased compared to the RI/R group and RI/R+shNC group (Figure 7C-D). Additionally, multi-labeled immunofluorescence revealed that IBA1^+^CCR2^+^ cells, IBA1^+^CCR2^-^MHC-II^+^ and IBA1^+^CCR2^+^MHC-II^+^ cells were decreased in the RI/R+LCN2 shRNA group compared to those in the RI/R group and RI/R+shNC group (Figure 7C, E-F). These findings indicate that the inhibition of LCN2 could alleviate antigen presentation in retinal microglia and macrophages following RI/R injury. We further found that C3 protein levels, C3 staining in the inner retina, and C3^+^/GFAP^+^ colocalized voxels were significantly decreased in the RI/R+LCN2 shRNA group compared to than those in the RI/R group and RI/R+shNC group (Figure 7G-K), suggesting that RI/R-induced complement activation was effectively suppressed by *Lcn2* knockdown.

### *Lcn2* knockdown ameliorated the excessive neuroinflammation following RI/R injury and exhibited a neuroprotective effect in retina

**Figure 8:**
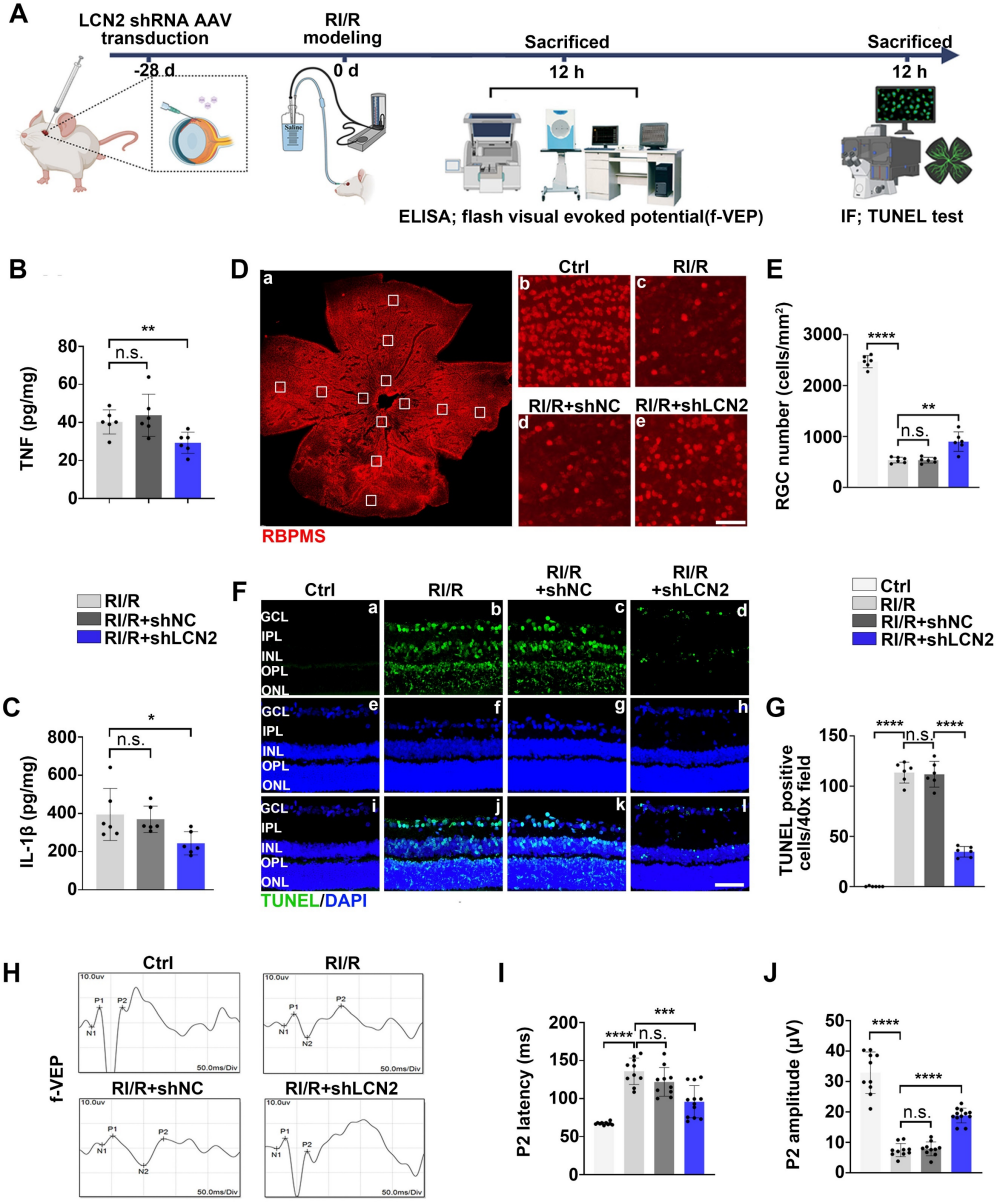
** LCN2 shRNA-AAV treatment down-regulated the expression of proinflammatory cytokines following RI/R injury and exhibited a neuroprotective effect on the retinae. A**: Schematic diagram of experimental workflow.** B, C**: ELISA showed the protein level of TNF and IL-1β in rat retinae following LCN2 shRNA-AAV treatment, compared to the RI/R group (n = 6). **D**: Immunofluorescence images showed the number of RBPMS-positive RGCs in rat retinae following LCN2 shRNA-AAV treatment, compared to the RI/R group. **E**: Bar graph depicted the number of RBPMS-positive RGCs per mm^2^ in each group (n = 6). **F**: TUNEL tests showed the apoptosis in rat retinae following following LCN2 shRNA-AAV treatment, compared to the RI/R group. **G**: Bar graph depicted the TUNEL positive cells in each group (n = 6). **H**: Visual function following RI/R with LCN2 shRNA-AAV treatment were measured by f-VEP test. **I**, **J**: Bar graphs depicted the amplitude of N2-P2 and the latency of P2 in each group (n = 6). Data in** B**,** C**,** E**,** G, I, J** were represented as mean ± SD; *p < 0.05, **p < 0.01, ***p < 0.001, ****p < 0.0001, n.s.: no significance, (compared with the control group/the RI/R group using Student t-test). Bar = 100 μm in **Db**-**De,** Bar = 50 μm in **Fa-Fi**. GCL: ganglion cell layer, IPL: inner plexiform layer, INL: inner nuclear layer, OPL: outer plexiform layer, ONL: outer nuclear layer.

Previous studies have shown that inflammatory responses can exacerbate neuronal damage and visual impairment after retinal diseases and injuries 58. Therefore, we examined the effects of *Lcn2* knockdown on RGC loss and visual impairment following RI/R injury (Figure 8A). ELISA assays revealed that *Lcn2* knockdown significantly reduced the up-regulation of TNF and IL-1β levels induced by RI/R injury (Figure 8B-C). Immunofluorescence analysis showed that the loss of RBPMS^+^ RGCs was significantly reduced by *Lcn2* knockdown (Figure 8D-E), and TUNEL assays revealed that *Lcn2* knockdown ameliorated retinal apoptosis (Figure 8F-G). F-VEP tests demonstrated that *Lcn2* knockdown significantly decreased the latency of P2 waves and increased their amplitudes (Figure 8H-J). Collectively, these findings suggest that *Lcn2* knockdown suppressed excessive neuroinflammation after acute glaucomatous damage and exerts a certain degree of neuroprotection in the retina.

### RNA-sequencing predicted the potential mechanisms of LCN2 in promoting neuroinflammation following RI/R injury

**Figure 9:**
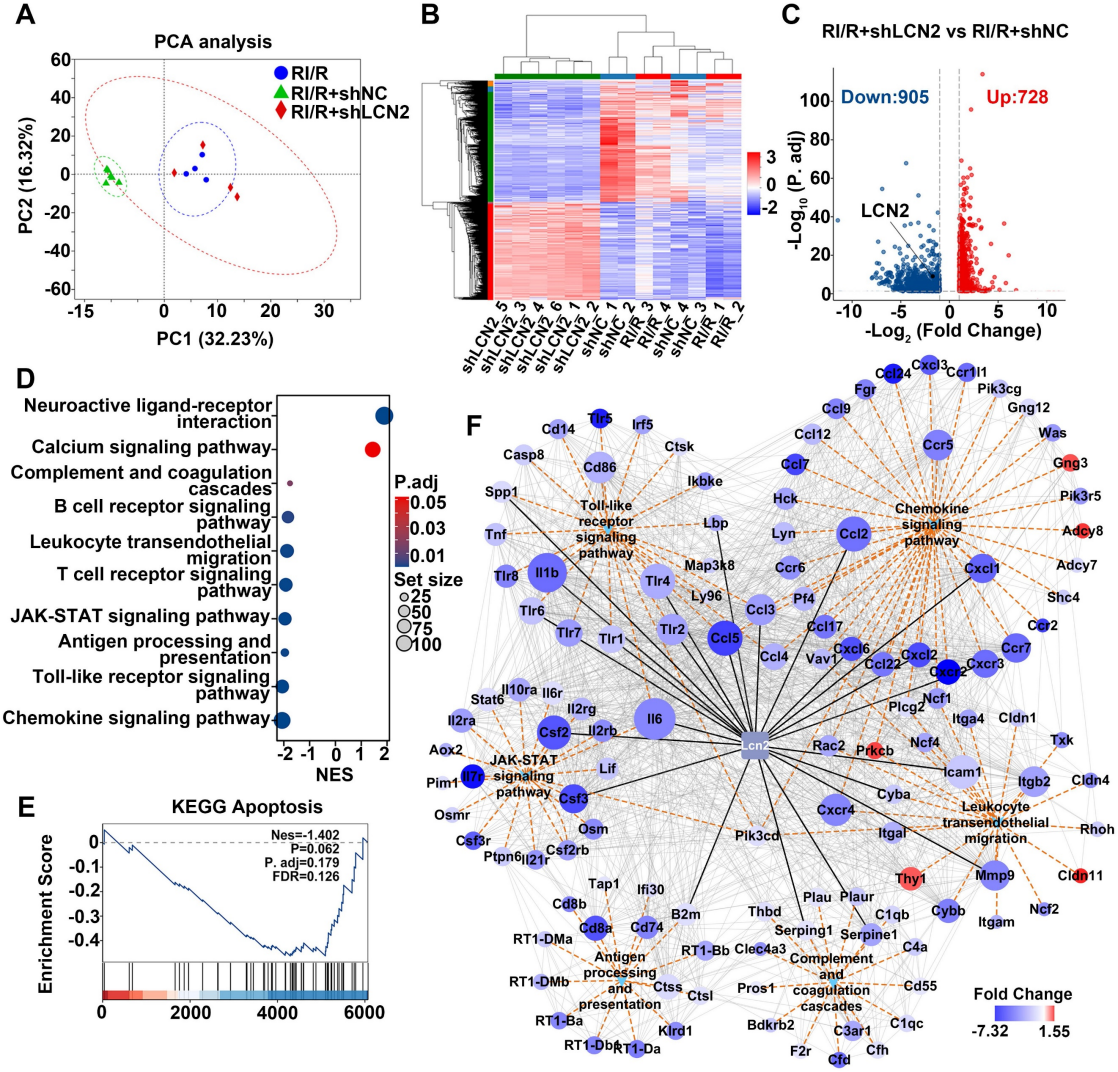
** Molecular mechanisms underlying *Lcn2* knockdown in RI/R retina. A**: PCA of retina samples from RI/R, RI/R+shNC and RI/R+shLCN2 groups (n = 4-6 per group). **B**: Heatmap and unsupervised hierarchical clustering of DEGs from RI/R, RI/R+shNC and RI/R+shLCN2 retinae. Scale represents z-score values of FPKM. **C**: Volcano plot shows DEGs in RI/R+shLCN2 versus RI/R+shNC group. **D**: GSEA for KEGG pathways. **E**: GSEA for Apoptosis pathways. **G**: The interaction diagram of genes belonging to the selected KEGG pathways: Chemokine signaling pathway, Leukocyte transendothelial migration, Antigen processing and presentation, Toll-like receptor signaling pathway, JAK-STAT signaling pathway and Complement and coagulation cascades. Orange dotted lines represent KEGG pathways. Grey solid lines represent genes-encoding protein interactions between different proteins. Black solid lines represent proteins directly interacted with LCN2. The size of nodes represents the number of protein interactions. The color bar from red to blue indicates the fold change of gene expression level from increasing to decreasing. NES: normalized enrichment score.

To explore the potential mechanisms by which LCN2 promotes neuroinflammatory damage following RI/R, we performed RNA-seq on retinae from RI/R, RI/R+shNC, and RI/R+shLCN2 rats. PCA and heatmap analysis revealed that the samples from the RI/R+shLCN2 group showed different transcriptomic profiles from those of the RI/R and RI/R+shNC groups (Figure 9A-B). A volcano plot displayed 728 up-regulated and 905 down-regulated genes following *Lcn2* knockdown treatment (Figure 9C). GSEA analysis of KEGG pathways demonstrated that pathways involved in antigen processing and presentation, inflammatory response (e.g., Toll-like receptor signaling pathway and JAK-STAT signaling pathway), peripheral infiltration (e.g., leukocyte transendothelial migration and chemokine signaling pathway), and complement and coagulation cascades were significantly down-regulated following *Lcn2* knockdown (Figure 9D). In contrast, pathways related to neuronal function (e.g., neuroactive ligand-receptor interaction pathway and calcium signaling pathway) were up-regulated (Figure 9D). Notably, we found that the apoptosis pathway was a trend of down-regulation in GSEA analysis, although this finding did not achieve statistical significance (Figure 9E). Next, we further investigated the PPI network between LCN2 and DEGs enriched in several down-regulated KEGG pathways. PPI results showed LCN2 interaction with genes in multiple signaling pathways including Toll-like receptor signaling pathway (*Il1β*, *Tlr2*, *Tlr4*, *Tlr6*, *Ccl3*, *Ccl5*, *Spp1*, *Lbp*); JAK-STAT signaling pathway (*Il6*, *Csf2*, *Csf3*); antigen processing and presentation (*B2m*); complement and coagulation cascades (*Serping1*, *Serpine1*); leukocyte transendothelial migration (*Icam-1*, *MMP9*, *Cyba*, *Cxcr4*, *Itgb2*); and chemokine signaling pathway (*Ccl2*, *Ccl3*, *Ccl4, Ccl5, Cxcl1, Cxcl2, Cxcl6, Cxcr2*) (Figure 9F).

Through intravitreal injection with LCN2 shRNA-AAV, qPCR tests revealed that the high transcriptional levels of *Tlr4* in the Toll-like receptor signaling pathway, *Il6* in the JAK-STAT signaling pathway, *Cxcl1*,* Cxc4* in the chemokine signaling pathway, *MMP9*, *Cxcr4*, *Itgb2* in the leukocyte transendothelial migration, *Serping1*, *Serpine1*, *C3ar1* in the complement and coagulation cascades, *B2m*, *Cd74* in the antigen processing and presentation following RI/R injury were significantly lower than those in the RI/R group and RI/R+shNC group (Figure S4B-P). These data illustrate that the potential mechanisms of LCN2 promoting neuroinflammatory damage following RI/R may be involved in the above multiple signaling pathways.

### Acute glaucomatous injury triggers the high expression of LCN2 in peripheral serum, which is seriously associated with the severity of neuroinflammatory response in retina

**Figure 10:**
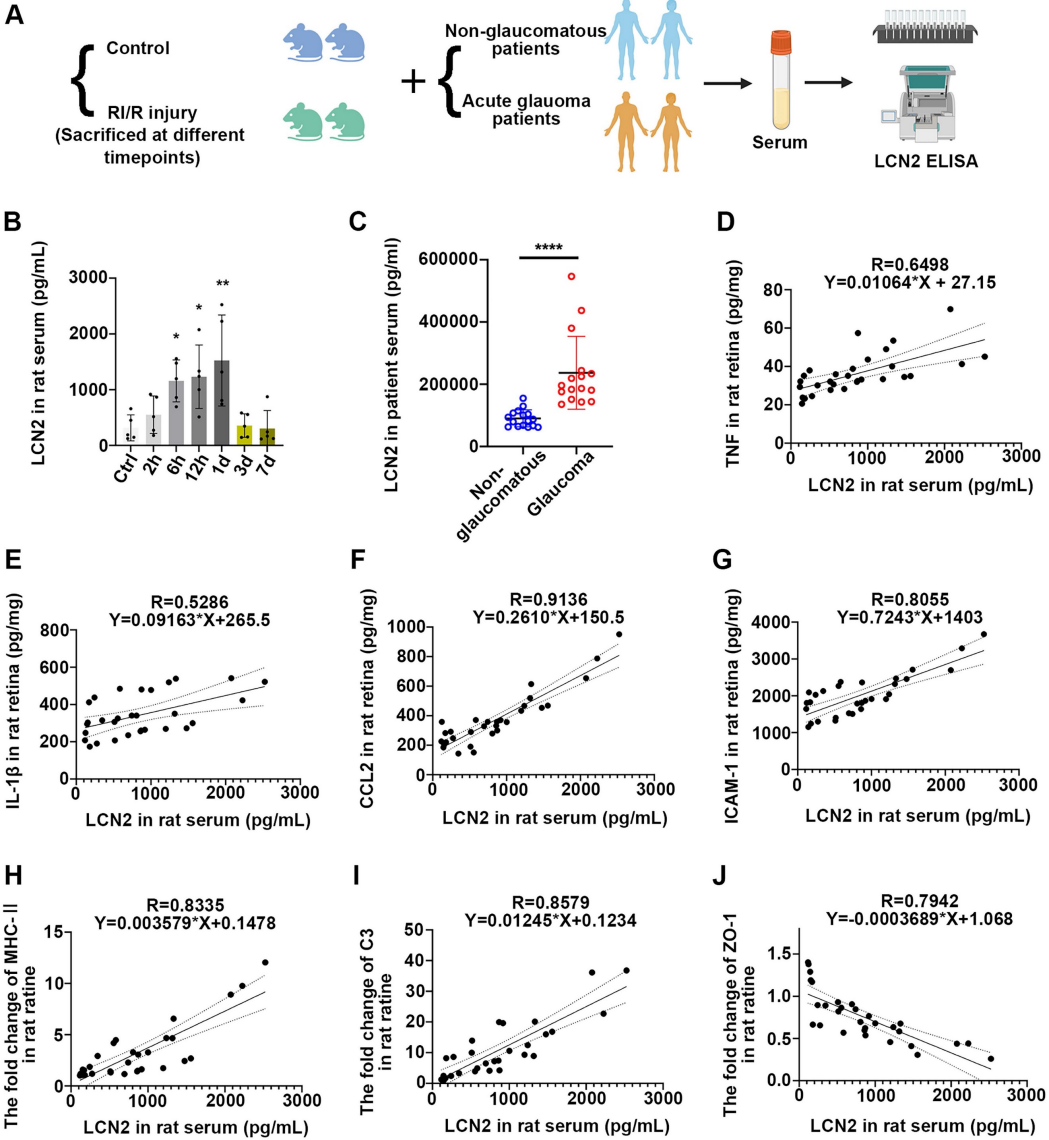
** Serum LCN2 was significantly up-regulated following acute glaucomatous injury and was associated with the severity of retinal neuroinflammatory response. A**: Diagram of serum sampling. **B**: ELISA showed the protein level of LCN2 in serum of rats following RI/R (n = 5). **C**: ELISA showed the protein level of LCN2 in serum of glaucomatous and non-glaucomatous patients (n = 16). **D-J**: Linear correlation between serum LCN2 protein levels and retinal TNF (**D**), IL-1β (**E**), CCL2 (**F**), ICAM-1 (**G**), MHC-Ⅱ (**H**), C3 (**I**) and ZO-1 (**J**) protein levels in RI/R rats. Data in **D**,** F-H** were represented as mean ± SD; *p < 0.05, **p < 0.01, ****p < 0.0001 (compared with the control group using one-way analysis of variance, compared with the non-glaucomatous group using Student t-test).

We further sought to determine the impact of acute high IOP-induced RI/R injury on the protein level of LCN2 in peripheral blood. Thus, we initially collected serum samples from rats with/without acute high IOP-induced RI/R injury (Figure 10A). ELISA showed that LCN2 expression was detectable at 6 h and reached its peak 1 d following RI/R (Figure 10B). The temporal pattern of LCN2 up-regulation in the peripheral serum of rats mirrored the pattern observed in the rat retina (Figure 5D-E). Subsequent analysis of serum from patients with/without acute glaucoma indicated that serum LCN2 levels were significantly elevated in individuals diagnosed with acute glaucoma compared to those in non-glaucomatous subjects (Figure 10A, C; Table S2). These data suggest that acute high IOP-induced RI/R injury can elicit a significant increase in LCN2 levels in the peripheral serum. Moreover, the temporal pattern of LCN2 up-regulation in the peripheral serum of rats closely coincided with the disruption of the BRB, infiltration of peripheral immune cells, and the innate immune cell response following RI/R injury, suggesting that the elevated expression of LCN2 in the peripheral blood may also be related to these events. Linear regression analysis was performed to test the correlation between the concentration of LCN2 in the rat serum and the expression of pro-inflammatory cytokines, chemokines, adhesion molecules, immune factors, and tight junction proteins. There were significant positive correlations between the concentration of LCN2 in rat serum and the expression of TNF, IL-1β, CCL2, ICAM1, MHC-II and C3 in rat retina (with TNF, R = 0.6498, *p* < 0.0001, R^2^ = 0.4222; with IL-1β, R = 0.5286, *p* < 0.0001, R^2^ = 0.2794; with CCL2, R = 0.9136, *p* < 0.0001, R^2^ = 0.8347; with ICAM1, R = 0.8055, *p* < 0.0001, R^2^ = 0.6489; with MHC-II, R = 0.8335, *p* < 0.0001, R^2^ = 0.6947; with C3, R = 0.8579, *p* < 0.0001, R^2^ = 0.7151) (Figure 10D-I). There was a negative correlation between the concentration of LCN2 in rat serum and the expression of ZO-1 in the rat retina (R = -0.7942, *p* < 0.0001, R^2^ = 0.6308; (Figure 10J). These findings imply that the concentration of LCN2 in peripheral serum is strongly associated with the severity of the neuroinflammatory response in the retina following acute glaucomatous injury.

## Discussion

### An interplay of peripheral immune cells and resident retinal glia following acute glaucoma triggers a “neuroinflammatory cascade” in retinal tissue

Besides the morphological alteration, the number of IBA1^+^ cells rapidly increased 12 h following RI/R and were mainly distributed in the superficial plexus of the retina (ganglion cell layer, inner plexiform layer, and inner nuclear layer) (Figure 3E, G). These layers are the primary distribution sites in the inner BRB 59. We found that the structure of tight junctions in the retina was disorganized 12 h after RI/R (Figure 2A-B, G-H). With the up-regulation of ICAM1 and CCL2, many CD45^+^ cells were distributed in the retinal tissue, and a considerable number of CD45^+^ cells accumulated near the gap of the retinal capillary walls, poised to penetrate the inner BRB into the retina (Figure 2G). Flow cytometry revealed that the simultaneous accumulation of mononuclear macrophages and lymphocytes was substantial, whereas there was no obvious accumulation of resident microglia (Figure 2I-K). These results suggested that the structure and function of the inner BRB were rapidly disrupted following acute glaucomatous damage, and the increased IBA1^+^CCR2^+^cells in the superficial plexus of the retinae (Figure 3E, G) implied the infiltration of peripheral macrophages. However, whether the infiltrating macrophages in the CNS are beneficial or detrimental to neuronal function remains debatable 60. Some studies have supported the idea that infiltrating macrophages mediate the clearance of apoptotic neurons and debris during stroke 61. Other studies have revealed that peripheral macrophages exacerbate OGD-induced neuronal death 62. Our fluorescent images demonstrated that ameboid IBA1^+^CCR2^+^ cells colocalized with MHC-II (Figure 3E, H), implying that the infiltrating macrophages participated in antigen presentation to the lymphocytes, consequently triggering retinal tissue-specific immune responses 62.

In addition, the activated microglia, as well as the reactive astrocyte and Müller cells, can activate the endothelial cells by pro-inflammatory cytokines (such as TNF-α, IL-1β, and IL-6) during retinal neuroinflammation. These activated endothelial cells up-regulate adhesion molecules and chemokines, thereby recruiting peripheral CD4^+^T cells 63. After secondary activation by antigen-presenting cells (such as resident activated microglia and infiltrating macrophages in the present study), these T cells breach the restriction of the BRB, infiltrate the retina, and primarily differentiate into Th1 cells 64. Previous study revealed that the IFN-γ produced by these infiltrating Th1 cells can conversely activate more resident retinal glia cells to exhibit pro-inflammatory effects 65. These studies fueled our hypothesis that there may be a complex interplay between peripheral immune cells and resident retinal glia cells, which could trigger a “neuroinflammatory cascade” following acute glaucomatous injury. To date, most studies have predominantly focused on individual immune components, with insufficient emphasis on summarizing their interactions. Understanding the precise roles of these immune cell populations and, as well as the mechanisms underlying their interactions during RI/R injury, is essential for exploring potential therapies that could mitigate inflammation and protect the retina from ischemia-reperfusion injury.

### LCN2 may be involved in neuroinflammatory damage following acute glaucoma through regulating multiple mechanisms

Previous studies have demonstrated that LCN2 promotes the expression and secretion of vascular endothelial growth factor A (VEGFA) in endothelial cells, leading to decreased expression of tight junction proteins, such as ZO-1, occludin, and claudin-5. These events result in blood-nerve barrier leakage and breakdown of neurovascular unit 66. Moreover, LCN2 can increase the pool of matrix metalloproteinase 9 (MMP9) and form a stable complex with MMP9, which reduces its degradation of MMP9 and further promotes BBB injury 67. Zadeh et al. revealed that VEGFA was up-regulated following RI/R 68, and Xu Zhang et, al. reported that MMP9 was activated during retinal ischemia 69. Additionally, LCN2 has been identified as an inducer of chemokine expression in the CNS 70. LCN2-induced chemokines act in an autocrine or paracrine manner to promote cell migration to inflammatory sites in the CNS, thereby perpetuating the neuroinflammatory response 71. In the current study, we found that genetic reduction of *Lcn2* significantly alleviated the disorganization of tight junctions and the up-regulation of ICAM1 and CCL2, thereby inhibiting peripheral immune cell invasion. Our PPI network analysis following *Lcn2* knockdown treatment predicted that *Icam-1*, *MMP9*, *Cyba*, *Ccl2*, *Ccl3*, *Ccl4, Ccl5, Cxcl1, Cxcl2, Cxcl6, Cxcr2*-encoded proteins interact with LCN2 (Figure 9F). Further ELISA and qPCR verified that *LCN2* knockdown suppressed the elevated ICAM-1 (Figure 6E), CCL2 (Figure 6F), *MMP9* (Figure S4I)*, Cxcl1* (Figure S4H) following RI/R injury. This evidence suggests that the LCN2-induced disruption of the inner BRB and infiltration of peripheral immune cells may be achieved by promoting MMP9, adhesion molecules, and chemokines.

Research has shown that LCN2 can activate two pro-inflammatory signaling pathways, TLRs-NF-κB and JAKs-STAT3, in glial cells to release neurotoxic products. LCN2 deficiency can inhibit the pro-inflammatory effects of glial cells after injury and rescue neuronal damage 56. Moreover, LCN2 expression is significantly elevated in retinal neuroinflammatory injuries 72. We found that LCN2 protein levels rapidly increased and peaked at 1 d following RI/R (Figure 5D-E). At this time point, an innate immune cell response (including antigen presentation and complement activation) was observed (Figure 3), coinciding with the up-regulation of pro-inflammatory cytokines (Figure S1B-C). The PPI network revealed that LCN2 interacted with molecules involved in antigen presentation and acute inflammatory response (Figure 5C). Administration of LCN2 shRNA-AAV resulted in the down-regulation of pro-inflammatory cytokines (Figure 8B-C) and the subsequent attenuation of an innate immune cell response (Figure 7). Previous studies have demonstrated that several classical pro-inflammatory signals, such as TrkB/Akt/NF-κB 73 and JAKs-STAT3 74, are activated following RI/R injury. Our bioinformatics analysis and qPCR tests indicated that some core genes in the Toll-like receptor and JAK-STAT signaling pathways were down-regulated following *Lcn2* knockdown treatment (Figure 9D, F; Figure S4B, D). These findings imply potential mechanisms through which LCN2 mediates pro-inflammatory effects following acute glaucomatous damage. In addition, we found that many MHC-II (e.g., *Cd74*, *RT1-DMa*, *RT1-DMb*, *RT1-Ba*, *RT1-Bb*, etc.) and MHC-I (*B2m*) protein-coding genes were down-regulated following *Lcn2* knockdown (Figure 9F). The PPI network indicated that the MHC-I component, B2M, was predicted to interact with LCN2 within the antigen processing and presentation pathways (Figure 9F). Our qPCR tests revealed that *LCN2* knockdown suppressed elevated *B2m* levels (Figure S4O). Although research on the role of B2M in ocular diseases is limited, neuroscience suggests that both B2M and LCN2 may serve as key biomarkers for assessing stroke risk and prognosis 75. Thus, the interaction between LCN2 and B2M in the context of antigen presentation after RI/R injury requires validation in future studies.

In addition to promoting neuroinflammation, LCN2 may exert direct toxicity in neurons. Liu et al. observed an increase in cleaved caspase-3 levels in wildtype neurons treated with LCN2, indicating that LCN2 promotes apoptosis 76. Our findings showed that knockdown LCN2 alleviated RI/R-induced apoptosis and suppressed the activation of apoptosis-related pathways (Figure 8F-G; Figure 9E). Further research is needed to determine whether the neuronal damage induced by LCN2 in this study is related to its direct toxicity.

### LCN2 in serum may invade into retina to exert pro-inflammatory effects and could serve as a potential biomarker

Up-regulation of LCN2 has been detected in the cerebrospinal fluid/blood of patients with ischemic stroke, suggesting that ischemic brain injury can trigger a high level of LCN2 not only at the site of damage, but also in the peripheral blood 77. In the current study, we observed an increase in LCN2 levels in both rat and human serum following RI/R injury (Figure 10A-C), implying that acute high IOP-induced RI/R injury triggers the up-regulation of LCN2 in the serum. Research on ischemic stroke has suggested that acute elevation of LCN2 in both blood and cerebrospinal fluid (CSF) are associated with neuroinflammation by promoting the infiltration of peripheral neutrophils and activation of pro-inflammatory glial cells 78-80. Wang et al. revealed that the intraperitoneal injection of an LCN2 monoclonal antibody attenuated neuroinflammation following brain ischemia/reperfusion injury 81. These findings emphasize the crucial role of peripheral LCN2 in neuroinflammation following ischemic injury. Considering that interference solely with intraocular LCN2 did not fully reverse RI/R-induced neuroinflammatory damage (Figure 8), the influence of peripheral LCN2 on retinal neuroinflammatory injury cannot be ignored.

Although some increased LCN2 at the ischemic site is thought to be derived from infiltrating neutrophils after transient middle cerebral artery occlusion 82, 83, we cannot confirm that the LCN2 observed in the infiltrating neutrophils is brought in from the periphery. Previous studies have revealed that LCN2 *in situ* can recruit peripheral neutrophils *in situ* and regulate their immune response by binding to receptor 24p3R on their cell membranes 77, 84, 85. Furthermore, the resident retinal glial cells can also crosstalk with peripheral immune cells, promoting them to release pro-inflammatory factors 86, 87. Accordingly, we cannot rule out the possibility that the increased LCN2 observed in infiltrating neutrophils within the brain is either bound to *in situ* LCN2 or be expressed in response to stimulation by local innate immune cells. In the future, it is imperative to analyze the cellular source of the elevated LCN2 in serum after acute glaucoma injury through comprehensive serum testing. Otherwise, it is worth noting that LCN2 in serum is primarily thought to stem from neutrophils 85, 88-90, and it's can be observed in numerous human tissues, including tubular cells in the kidney, heart, lung, liver, stomach, colon, epithelial cells, macrophages, dendritic cells and adipocytes 91. This evidence has guiding significance for future exploration of the source of elevated LCN2 levels in peripheral blood following acute glaucoma injury. It is also essential to investigate whether peripheral LCN2 directly affects retinal inflammation in acute glaucomatous injury directly by being transported to the injured site through the compromised inner BRB, or indirectly by modulating peripheral immunity. This issue warrants further exploration.

Furthermore, linear regression analysis revealed that the concentration of LCN2 in the peripheral serum was positively correlated with the expression of pro-inflammatory cytokines, chemokines, adhesion molecules, and the disorganization of tight junctions (Figure 10D-J). These results suggest that the concentration of LCN2 in the peripheral blood may reflect the extent of tight junction disruption in the neurovascular unit, the increase in pro-inflammatory and immune factors, and the degree of peripheral immune cell infiltration under acute glaucoma conditions, thereby predicting the severity of retinal neuroinflammatory damage. Should future comprehensive clinical studies validate the correlation between LCN2 and retinal neuroinflammation in patients with acute glaucoma, LCN2 may serve as a promising biomarker for predicting the severity of this condition.

## Conclusions

In conclusion, our data revealed the breakdown of the inner BRB, along with peripheral immune cell infiltration and innate immune cell responses, during the early stages of acute glaucomatous injury. These events likely created a positive feedback circuit that exacerbated the inflammatory cascades involved in retinal glaucomatous damage. We also identified that *Lcn2* knockdown could simultaneously ameliorate the critical elements of the aforementioned “inflammatory cascades”, thereby exhibiting a neuroprotective effect in the retina. Based on these data and in-depth analysis of animal and human serum, we proposed that LCN2 potentially served as a candidate biomarker for predicting the severity of acute glaucoma. Our study identified a novel mechanism of LCN2 in Acute high IOP induced neuroinflammation and may provide new evidences to retinal repair strategies for better visual function recovery on intervention time points and new targets.

## Supplementary Material

Supplementary figures and tables.

## Abbreviations

IOP: intraocular pressure
RGCs: retinal ganglion cells
RI/R: retinal ischemia reperfusion
OGD/R: oxygen-glucose deprivation/reoxygenation
AAV: adeno-associated virus
DAMPs: damage-associated molecular patterns
BRB: blood-retinal barrier
BNB: blood-nerve barrier
RT: room temperature
GO: Gene Ontology
KEGG: kyoto encyclopedia of genes and genomes
GSEA: gene set enrichment analysis
DEGs: differential expression genes
PCA: principal component analysis
PPI: protein-protein interaction
EB: Evans Blue
MHC-II: major histocompatibility complex class II
C3: complement C3
LCN2: lipocalin-2
f-VEP: flash visual evoked potential
CSF: cerebrospinal fluid
VEGFA: vascular endothelial growth factor A
MMP9: matrix metallopreoteinase 9
tMCAO: transient middle cerebral artery occlusion
GCL: ganglion cell layer
IPL: inner plexiform layer
INL: inner nuclear layer
OPL: outer plexiform layer
ONL: outer nuclear layer
ROI: region of interest
