# Supplementary material for: LCN2 deficiency mitigates the neuroinflammatory damage following acute glaucoma
Source: Theranostics. 2025 Feb 10;15(7):2967–90. doi: 10.7150/thno.104752 (PMC11898297; doi:10.7150/thno.104752)
Supplement: Supplementary file 1 — Supplementary figures and tables. [file thnov15p2967s1.pdf]

LCN2 deficiency mitigates the neuroinflammatory damage following acute glaucoma

Tu Hu[a,b,c,1], Shuhan Meng[a,b,c,1], Can Liu[d], Weizhou Fang[a,b,c], Zhaohua Xia[a,b,c], Yiqun Hu[e], Jia Luo[f], Xiaobo Xia[a,b,c,*]

[a] *Eye Center of Xiangya Hospital, Central South University, Changsha, Hunan, China 410008*

[b] *Hunan Key Laboratory of Ophthalmology, Changsha, Hunan, China 410008*

[c] *National Clinical Research Center for Geriatric Disorders, Xiangya Hospital, Central South University, Changsha, Hunan, China 410008*

[d] *Department of Anatomy and Neurobiology, School of Basic Medical Sciences, Central South University, Changsha, Hunan, China, 410013*

[e] *Xiangya Medical School, Central South University, Changsha, Hunan, China, 410013.*

[f] *The First Clinical college, Changsha Medical University, Changsha, Hunan, China, 410203.*

***Corresponding author:**

Xiaobo Xia: Email xbxia21@csu.edu.cn

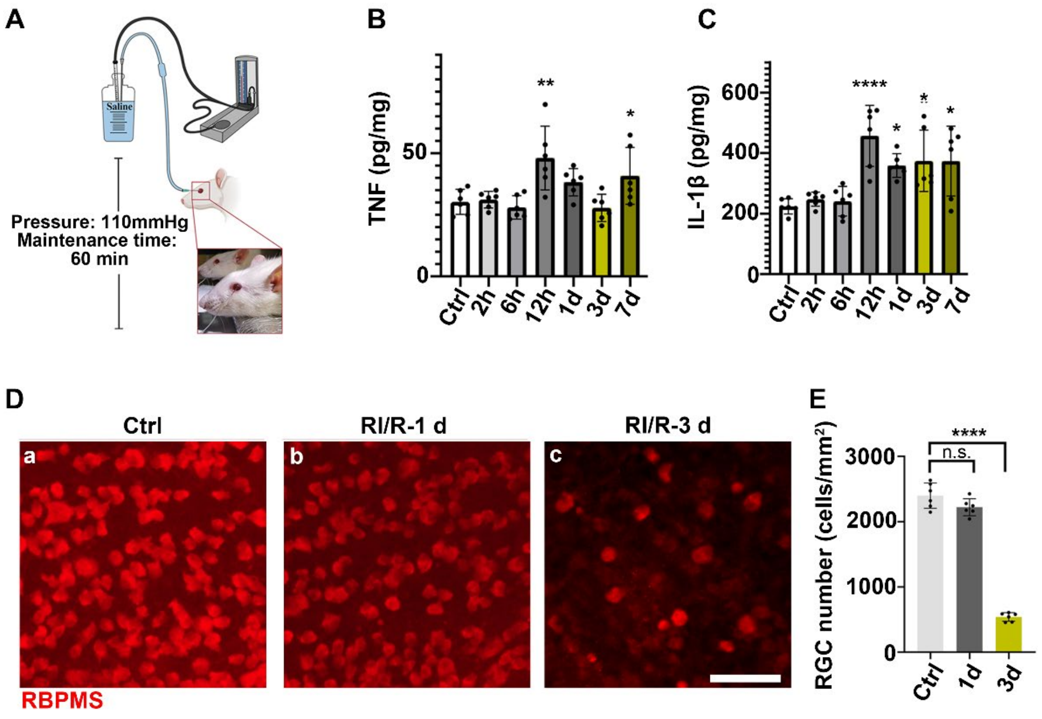

Figure S1. Inflammatory response occurred prior to RGC loss following RI/R injury. A: Diagram of RI/R model. **B**, **C**: ELISA showed the protein levels of TNF and IL-1β in rat retinae following RI/R injury, compared to the control group (n = 6). **D**: Immunofluorescence images showed the number of RBPMS positive-RGCs in rat retinae following RI/R injury, compared to the control group. **E**: Bar graph depicted the number of RBPMS positive-RGCs per mm^2 in each group (n = 6). Data in **B**, **C**, **E** were represented as mean ± SD; *$p < 0.05$, **$p < 0.01$, ****$p < 0.0001$, n.s.: no significance, (compared with the control group using one-way analysis of variance). Bar= 100 μm in **D**.

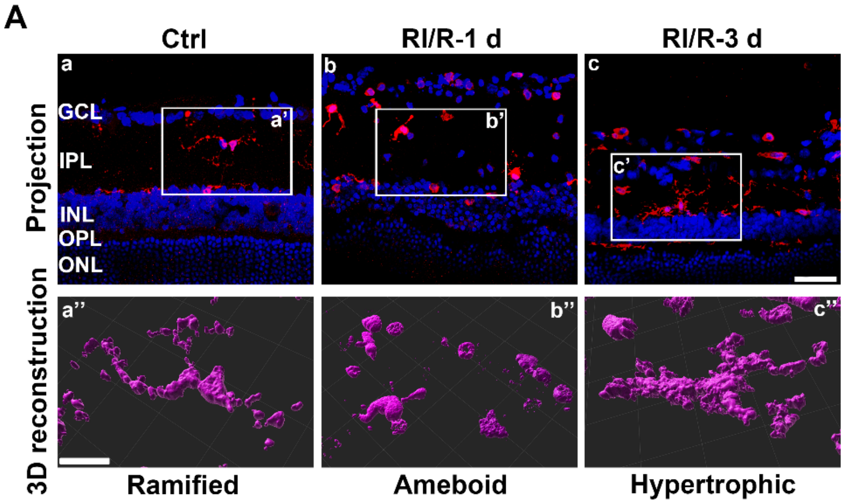

Figure S2. Dynamic changes in IBA1 positive cell morphology following RI/R. A: Representative 3D reconstruction of retinal IBA1-postive cell morphology following RI/R. Ramified shape characterized by the small cell body volume and long thin processes, which enabled microglia to monitor the surrounding environment. Ameboid morphology characterized by round sphere shapes and few processes, which was associated with microglia/macrophage phagocytosis and migration during disease and injury. Hypertrophic morphology characterized by enlarged cell body (compared to ramified shape) and fewer, shorter hypertrophi*c* processes, which represented a post-injury morphology.

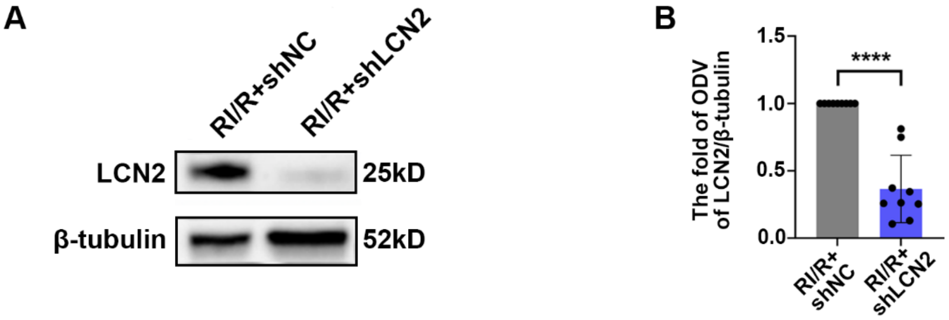

Figure S3. LCN2 shRNA-AAV knocked down retinal LCN2 expression following RI/R. A: Western blotting showed the change of LCN2 expression in rat retinae with the LCN2 shRNA-AAV treatment following RI/R. **B**: Bar graphs depicted the fold of ODV of LCN2 compared to the RI/R+shNC group (n = 9). Data in **B** were represented as mean ± SD; $****p < 0.0001$ (compared with the RI/R+shNC group using Student t-test).

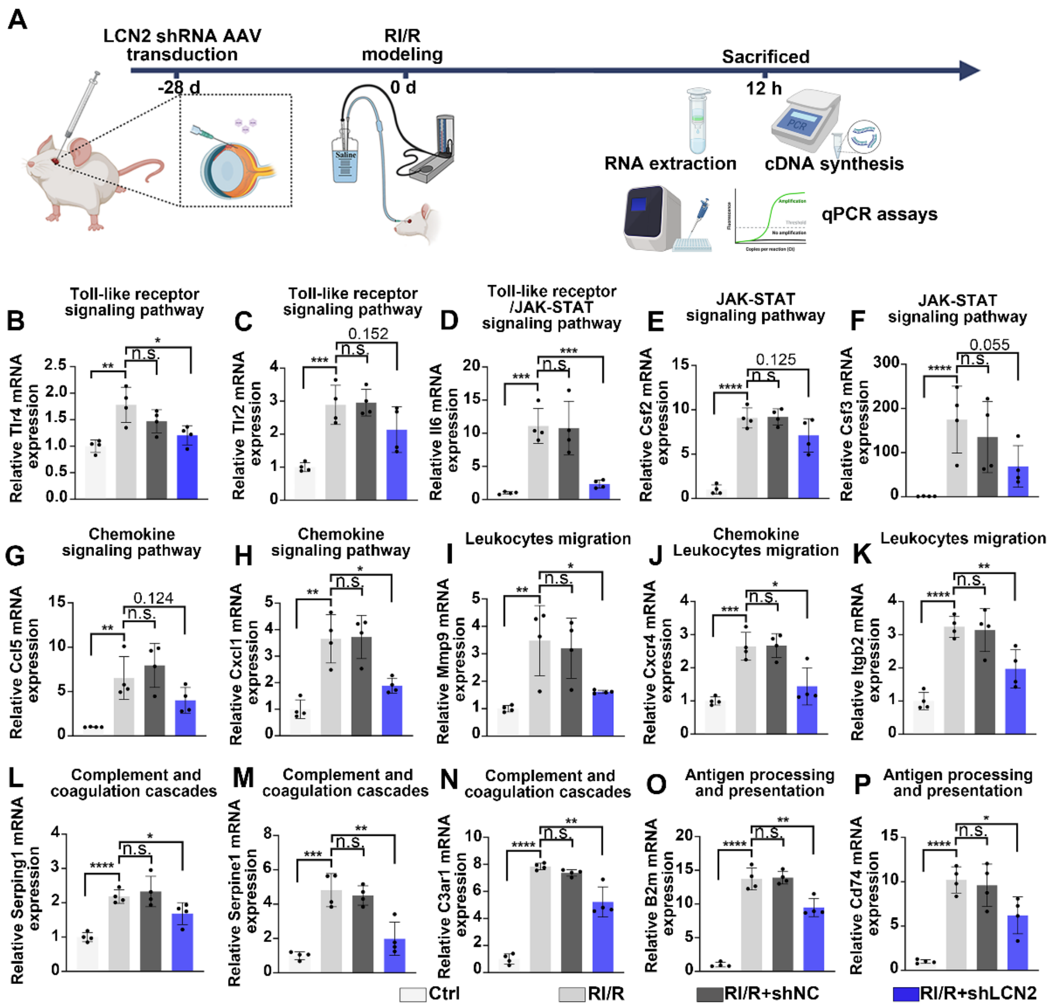

Figure S4. qPCR verification of key genes affected by *Lcn2* knockdown following RI/R. A: Diagram represented workflow of qPCR analysis. **B-P**: Bar graphs depicted the relative expression of target mRNA in each group (n = 4). Data in **B-P** were represented as mean ± SD; **p* < 0.05, ***p* < 0.01, ****p* < 0.001, *****p* < 0.0001, n.s.: no significance, (compared with the RI/R group using Student t-test).

Table S1. Primer sequences for qPCR analysis

Gene	Forward	Reverse
Tlr4	5'-TTGCTGCCAACATCATCCAGGAAG-3'	5'-CAGAGCGGCTACTCAGAAACTGC-3'
Tlr2	5'-TCTGGAGTCTGCTGTGCCCTTC-3'	5'-GGAGCCACGCCCACATCATTC-3'
Il6	5'-ACTTCCAGCCAGTTGCCTTCTTG-3'	5'-TGGTCTGTTGTGGGTGGTATCCTC-3'
Csf2	5'-AATGACATGCGTGCTCTGGAGAAC-3'	5'-TGGTGAGGTTGCCCCGTAGAC-3'
Csf3	5'-GGCTCTTCCTCTACCAAGGTCTCC-3'	5'-AGATGGTGGTGGCAAAGTTGTCG-3'
Ccl5	5'-GACACCACTCCCTGCTGCTTTG-3'	5'-CTCTGGGTTGGCACACACTTGG-3'
Cxcl1	5'-GCAGACAGTGGCAGGGATTCAC-3'	5'-AGTGTGGCTATGACTTCGGTTTGG-3'
Mmp9	5'-CTCCTGGTGCTCCTGGCTCTAG-3'	5'-GCTGTGTGTCCGTGAGGTTGG-3'
Cxcr4	5'-AAGCAAGGATGTGAGTTCGAGAGC-3'	5'-CCGAGGAAGGCGTAGAGGATGG-3'
Itgb2	5'-TTGCAGCAGAAGGACGGAAACG-3'	5'-ATGACCAGGAGGAGGACACCAATC-3'
Serping1	5'-GGCGGAGAACACCAACCACAAG-3'	5'-TTGGCACTCAAGTAGACGGCATTG-3'
Serpine1	5'-GCGTCTTCCTCCACAGCCATTC-3'	5'-TGTCTCTGTTGGATTGTGCCGAAC-3'
C3ar1	5'-GCCTTGAGAGAAGTTGTGCTACCC-3'	5'-AATGCCCTTTACGGACTGTCTTGC-3'
B2m	5'-ACCGTGATCTTTCTGGTGCTTGTC-3'	5'-ACACGTAGCAGTTGAGGAAGTTGG-3'
Cd74	5'-TCCTGGTGGCTCTGCTCTTGG-3'	5'-TCATGCGAAGGTTCTCCAGTTGC-3'
Gapdh	5'-AGTGCCAGCCTCGTCTCATA-3'	5'-GACTGTGCCGTTGAACTTGC-3'

Table S2. Demographic data and clinical characteristics of patients.

	non-glaucomatous	glaucoma
n	16	16
median age; years	65 (49-74)	69 (51-77)
female sex	56.3% (9)	62.5% (10)
disease type	age-related cataract	acute primary angle closure glaucoma
diabetes	-	-
hypertension	-	-
